# Age, but not an immune challenge, triggers terminal investment in the Pacific field cricket, *Teleogryllus oceanicus*

**DOI:** 10.1093/beheco/arad021

**Published:** 2023-03-30

**Authors:** Nicola-Anne J Rutkowski, Yong Zhi Foo, Therésa M Jones, Kathryn B McNamara

**Affiliations:** School of BioSciences, University of Melbourne, Biosciences 4, Royal Parade, Parkville, Victoria 3010, Australia; Centre for Evolutionary Biology, School of Biological Sciences, University of Western Australia, 35 Stirling Hwy, Crawley, Western Australia 6009, Australia; School of BioSciences, University of Melbourne, Biosciences 4, Royal Parade, Parkville, Victoria 3010, Australia; School of BioSciences, University of Melbourne, Biosciences 4, Royal Parade, Parkville, Victoria 3010, Australia

**Keywords:** ecological immunology, reproductive plasticity, reproductive trade-offs, residual reproductive value

## Abstract

The terminal investment hypothesis proposes that, when individuals are faced with a threat to survival, they will increase investment in current reproduction. The level of the threat necessary to elicit terminal investment (the dynamic terminal investment threshold) may vary based on other factors that also influence future reproduction. Here, we tested whether there is an interactive effect of age and an immune challenge on the dynamic terminal investment threshold in the Pacific field cricket, *Teleogryllus oceanicus*. We measured the courtship call, mating attractiveness, ejaculate size, and offspring production of *T. oceanicus* males. We found only limited support for the dynamic terminal investment threshold: there was no consistent evidence of a positive interaction between male age and immune challenge intensity. However, we found evidence for age-related terminal investment: older males produced a larger spermatophore than younger males. Older males also had a slower calling rate compared to younger males, suggesting a potential trade-off between these two pre- and post-copulatory traits. As some, but not all, reproductive traits responded plastically to cues for terminal investment, our research highlights the importance of considering a broad range of pre-and post-copulatory traits when exploring the potential for terminal investment to occur.

## Teaser

Terminal investment theory suggests that when time is running out, or if you are really sick, you should go all-in on reproduction. In the Pacific field cricket, we show that, while older males produce heavier ejaculates than their younger counterparts, their immune status has little effect on their reproductive investment. Thus, we demonstrate that Pacific field crickets do show terminal investment, but in only one of a suite of pre- and post-copulatory reproductive traits.

## INTRODUCTION

Organisms have a limited reservoir of resources and are unable to maximize investment into all life-history traits concurrently (Sinervo and Svensson 1998; Zera and Harshman 2001). As a result, trade-offs among life-history traits are ubiquitous (Stearns 1989; Zera and Harshman 2001). One fundamental trade-off is that between current and future reproduction (Stearns 1989; Ghalambor and Martin 2001), which is dependent not only on the individual’s condition, but also their predicted lifespan. With each successful reproductive event, the likelihood of surviving to the next decreases (Williams 1966). Therefore, individuals with low residual reproductive value (RRV; an individual’s expectation for future progeny), could maximize their fitness by investing relatively more in the current reproductive event, despite the likelihood that this may decrease their longevity. Individuals with high RRV should do the opposite, allocating more to future reproduction instead. This phenomenon, called the “terminal investment” hypothesis (Clutton-Brock 1984), has been empirically supported in a range of taxa (for an extensive review of terminal investment, see Duffield et al. [2017]). However, how specific factors (such as age or an immune challenge) may interact to alter an individual’s RRV, and therefore the threshold for terminal investment, remains poorly understood.

An important theoretical prediction of the terminal investment hypothesis is that the switch in investment is dependent on an individual’s RRV. Intrinsic factors, such as age, have the potential to affect an individual’s RRV. Typically, RRV is expected to decrease with age, and evidence for age-dependent terminal investment has been found in males and females in both vertebrates (Velando et al. 2006; Descamps et al. 2007) and invertebrates (Shoemaker et al. 2006; Lafaille et al. 2010; Heinze and Schrempf 2012; Gonzalez-Tokman et al. 2013; Farchmin et al. 2020). However, a decrease in RRV with age is not universal, and there is evidence that it can be compensated for in some taxa, via age-related learned foraging behaviors (Desrochers 1992; Froy et al. 2015) or improvements in reproductive effort (Curio 1983).

A number of extrinsic factors can also affect RRV, either by directly influencing reproduction (e.g., mate availability; Duffield et al. 2017) or indirectly by altering the perceived probability of survival (Bonneaud et al. 2004). One key extrinsic factor that has received substantial empirical attention is infection (Adamo 1999; Sadd et al. 2006; Cotter et al. 2011; Hendry et al. 2016; Miyashita et al. 2019; Jehan et al. 2021). Traditionally, it was believed that when individuals face a threat to their survival, they should transfer investment away from reproduction and toward recovery and defense (thereby investing in future reproductive opportunities) (Svensson et al. 1998; Adamo et al. 2001; Jacot et al. 2004). However, increasing evidence suggests that some infected organisms instead increase their investment in reproduction, consistent with the terminal investment hypothesis (Bonneaud et al. 2004; Sadd et al. 2006; Hendry et al. 2016). Studies manipulating an individual’s perceived survival via an immune challenge have typically used a single dose of a parasite or pathogen (Adamo 1999; Nielsen and Holman 2012). More recently, papers have manipulated variation in the intensity of immune challenges (Copeland and Fedorka 2012; Hendry et al. 2016; Duffield et al. 2018; Duffield et al. 2019; Duffield et al. 2020). For example, in the pea aphid, *Acyrthosiphon pisum*, reproductive output increased only after a high, but not a low, dose of a live pathogen (Hendry et al. 2016). Together, these results provide support that the intensity of the cue (e.g., the pathogen dose) can be viewed as a threshold trait for eliciting terminal investment. Inactive immune elicitors (such as heat-killed bacteria, lipopolysaccharides [LPS], Sephadex beads, or peptidoglycans [PGN]), which mimic a natural infection, without the confounding effects of physiological sickness (Milutinovic and Kurtz 2016), have also been used to stimulate an immune response and decouple host responses to perceived infection or infection risk.

The switch to increase or decrease reproductive investment exists on a continuum, where individuals reduce investment when RRV is high, and terminally invest when it is low. It is increasingly clear that both intrinsic and extrinsic factors have the ability to affect an individual’s RRV and are likely to act in concert. Recent theoretical models suggest that the intensity of the cue required to elicit terminal investment is context-dependent and dynamic (Duffield et al. 2017). These dynamic terminal investment threshold models suggest that individuals have a baseline RRV, and any further intrinsic or extrinsic factors acting on an individual can alter this threshold for terminal investment but may not result in death. For example, age may modify the intensity of the second cue that is required to elicit terminal investment (Duffield et al. 2017). Due to the difference in RRV between young and old individuals, the intensity of a terminal investment trigger, such as the strength of an immune challenge, should be lower for older individuals. Whereas younger individuals will require a higher dosage to elicit terminal investment. Here, terminal investment is defined as an increase in reproductive investment which exceeds the baseline (i.e., the mean value for the control treatment).

Several studies have looked at the impact of age and immune challenge on terminal investment, with some finding evidence of an interactive effect on the terminal investment threshold. For example, in the blue footed booby, *Sula nebouxii*, both the number of hatchlings and fledglings was higher for older males challenged with an immune elicitor compared with a control (Velando et al. 2006). Similarly, young male decorated crickets, *Gryllodes sigillatus*, immune challenged with heat-killed *Escherichia coli*, reduced calling effort at all levels of a bacterial infection, whilst older males increased their calling effort at moderate and high dosages, suggesting that only the older males may be terminally investing (Duffield et al. 2018).

A potential criticism of terminal investment studies is that they frequently only assess one pre- or post-copulatory trait as a measure of an individual’s reproductive investment (Jacot et al. 2004; Barribeau et al. 2010). However, the direction of impact of one trait might not be comparable for all others, especially when trade-offs are expected between different reproductive traits, e.g., between pre- and post-copulatory traits (Simmons et al. 2017). Therefore, by measuring single traits in isolation, studies may fail to detect the overall impact of terminal investment on sexual selection. Indeed, it is unclear if terminal investment differentially affects pre- or post-copulatory reproductive investment. For example, a study on the southern ground cricket, *Allonemobius socius* examined the acoustic signaling and mating success of males challenged with different doses of an immune elicitor. At higher doses, older males exhibited terminal investment in acoustic signaling, with the opposite pattern observed for younger males. However, there was no effect on mating success, demonstrating that not all pre-copulatory reproductive behaviors respond comparably (Copeland and Fedorka 2012). This may be because traits vary in their response to a reduced RRV (Duffield et al. 2017), and highlights the importance of considering the plasticity of multiple reproductive traits.

The Pacific field cricket, *Teleogryllus oceanicus*, is an ideal species to explore dynamic thresholds of terminal investment, as their pre- and post-copulatory traits, and how these covary with immunocompetence, have been well-studied (Simmons and Roberts 2005; Simmons 2012; McNamara et al. 2014). Males produce multi-modal sexual signals to attract females (acoustic and chemical) (Simmons et al. 2013b). The acoustic signal is comprised of both a long-range mate attraction call (the advertisement call), and a short-range call to elicit copulation (courtship call) (Hack 1997; Zuk et al. 2008). Female *T. oceanicus* prefer courtship calls with higher duty cycles (higher rates of call syllable production) (Simmons et al. 2013b) and longer calls (Rebar et al. 2009). And, importantly, male acoustic signals correlate with immunocompetence (Tregenza et al. 2006; Simmons et al. 2010), suggesting that calls are susceptible to changes in immune function. Male crickets also produce a spermatophore, a proteinaceous container filled with sperm and seminal fluid proteins (Simmons 2012; Sturm 2014). Male post-copulatory investment (ejaculate quality) in *T. oceanicus* correlates with immunity, both genetically and phenotypically (Simmons and Roberts 2005), which is supported by the reduction in male sperm viability following an experimental immune challenge (Simmons 2012).

We examined the effect of male age and immune status on terminal investment strategies, to test for evidence of a dynamic terminal investment threshold. Here, we provided young or old adult male *T. oceanicus* with immune challenges of increasing strength, and then quantified male pre-copulatory (courtship call and mating attractiveness) and post-copulatory (ejaculate size) investment. We predicted, given the theoretical expectations of the dynamic threshold model that older males would exhibit terminal investment, in the form of increased reproductive effort, at lower dosages of a bacterial challenge, whilst the opposite would be true for younger males. Finally, although theoretical and empirical studies demonstrate trade-offs between pre- and post-copulatory traits (Simmons et al. 2010; Mehlis et al. 2015; Simmons et al. 2017), it is not clear whether such trade-offs should be expected under terminal investment.

## METHODS

### Stock population

Experimental crickets were obtained from a large out-bred laboratory population (>1000 individuals) of Pacific field crickets (*Teleogryllus oceanicus)* originally collected from Carnarvon, Western Australia. Individuals were reared in large containers (25 × 14 × 17 cm) containing egg cartons for shelter, ad libitum water and cat chow and held at a constant temperature (25 °C) on a 12-h light: 12-h dark photoperiod. Experiments were conducted under a red-light, to simulate darkness (Schaefer and Wilkinson 2004).

### Age and immune status treatments

To examine simultaneously the effect of male age (and RRV) and immune challenge on terminal investment strategies, male stock penultimate-instar crickets were isolated in individual plastic containers (7 × 7 × 5 cm) with ad libitum cat chow and water. On the day of adult eclosion, males were haphazardly assigned to one of two treatments which manipulated the age at which they were exposed to an immune challenge. “Young” males (n = 90 males) received the challenge 10 days after adult eclosion (the approximate age at which males become sexually mature; Simmons 2012); “old” males (*n* = 108 males) received the challenge 20 days after adult eclosion (which is approximately 75% of the average adult lifespan in the laboratory stock population [N.R., personal observation]). Within the two male age treatments, males were allocated to receive one of four doses of an immune challenge treatment. First, already formed spermatophores (if present) were removed from the male’s genital pouch. Then, males were haphazardly assigned to receive a lipopolysaccharide (LPS) (*Serratia marcescens;* Sigma-Aldrich L6136) solution containing either: 0% (Control), 0.1%, 0.5%, or 1% LPS. The LPS was dissolved in 10 µL of phosphate-buffered saline and injected intra-abdominally with a micro-syringe (2000; SGE Analytical Science). The needle was between the second and third segment on the cricket’s ventral side. LPS is a component of the bacterial cell wall that induces an immune response (mimicking a bacterial infection), without the confounding effects of physiological sickness (Duffield et al. 2017). The LPS doses were selected based on the literature for comparable invertebrate studies, controlling for body size (Copeland and Fedorka 2012; Simmons 2012). LPS dosages were made from a single vial. We recorded male mortality due to age and LPS dose. All subsequent assays were conducted blind to the experimental treatment.

### Male reproductive investment

Twenty-four hours after his immune challenge, we assayed four measures of male pre- and post-copulatory reproductive investment: male courtship call, mating behavior, offspring production, and spermatophore weight.

To assess the effect of an immune challenge and age on courtship call and mating behavior, a virgin experimental male was paired with a virgin 10-day-old stock female. The male was placed in a small plastic container (14 × 10 × 7 cm) which was lined with soundproofing foam (4.5 cm thick). The female was placed in a small mesh-enclosed container (7 × 6 cm) inside the male’s container, prohibiting physical contact, but allowing transfer of visual and chemical cues. Courtship calls were recorded using a Digitech digital voice recorder (XC-0383). After 2 min, the female was released from the mesh container and the pair were permitted to court and mate. Pairs were given 30 min to mate and were observed for the duration, or until a successful mating had taken place. If a male did not mate within this time, he was given a new female for an additional 30 min (courtship call was not recorded). If a male did not mate after this period, he was returned to his rearing container and recorded as unmated. For all trials, we recorded the latency until calling, the total time spent calling and the structure of the call (see below). Additionally, we measured the time taken for the female to mount the male, and the time for the spermatophore to be transferred.

To assess whether male age or immune challenge affected male offspring production, mated females were transferred to a new container (7 × 7 × 5 cm), containing ad libitum water, cat chow and a Petri dish (4 × 1.5 cm) containing wet sand for oviposition (a sand pad). Eggs were collected every 4 days for a total of 8 days. Eggs were rinsed from the sand, counted, and placed onto a moist cottonwool pad following Jones et al. (2015). Eggs were assessed daily for 3 weeks for signs of hatching. The total number of eggs laid, and number of eggs hatched was recorded.

Next, we assessed the impact of immune challenge and age on male spermatophore weight (wet mass). Three hours after the completion of the courtship call and mating behavior assay, each male was given 30 min to mate with a 10-day-old stock female. The spermatophore (the second produced by each male) was removed immediately from the female (the sperm-containing ampulla of the spermatophore is attached externally to the female) and weighed to the nearest 0.01 mg on a Mettler Toledo balance (XS205). Spermatophore weight was highly repeatable across consecutive measurements of an individual spermatophore (ρ = 0.95, *n* = 9, *P* < 0.001). If a male did not mate within 30 min, the male was presented with a novel female for an additional 30 min. Males that did not mate were recorded.

Finally, all males were returned to their containers, provided with ad-libitum food and water and checked daily for survival.

### Bioacoustic analysis

Variation in male courtship calls was quantified using Audacity (v.2.4.2; Audacity team, 2021). Each recording was first filtered to remove background noise at <3.5 kHz following Simmons et al. (2013b), and two measures related to courtship calling were assessed: (1) call structure and (2) male calling investment.

**Figure 1:**
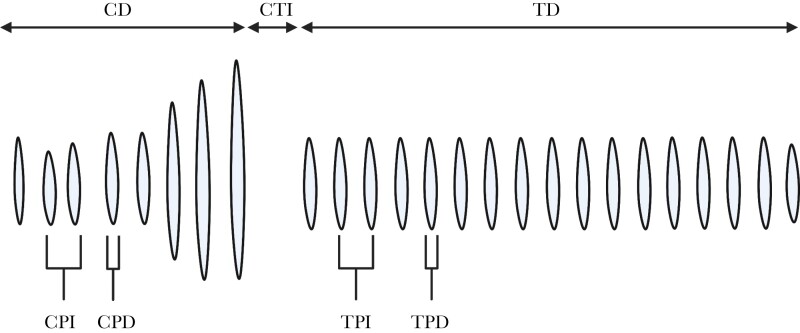
Waveform of a single artificially-constructed courtship call of *Teleogryllus oceanicus*. CD = chirp duration, CPI = chirp pulse interval, CPD = chirp pulse duration, CTI = chirp-trill interval, TD = trill duration, TPI = trill pulse interval, TPD = trill pulse duration. Modified from Simmons et al. (2013b).

The courtship call of *T. oceanicus* consists of two parts, the chirp and trill. These are both comprised of a series of pulses, where each pulse corresponds to a single wing closure. We haphazardly selected four consecutive courtship calls for each male. For each call, we quantified nine parameters, known to impact female mate choice following Simmons et al. (2013b) (Figure 1).

For CPI, we measured the mean interval between the first and second pulses and the second and third pulses of the chirp, and for CPD, we measured the first and second pulses in the chirp. Similarly, for TPD, we calculated the mean of the fifth and sixth pulses of the trill. For TPI, we calculated the mean interval between the fifth and sixth and the sixth and seventh pulses.

Additionally, we calculated the rate of courtship calls from the first 2 min of calling (regardless of whether this was before or after males and females were allowed physical contact). Here, we calculated the number of bouts (defined as a section of repetitive calls with a less than 5-s pause between calls) within the 2 min, the duration of each bout, the number of calls within each bout, and the inter-bout interval. We were then able to calculate the overall rate of calling over the 2-min period.

### Data analysis

Data were analyzed using R (v1.1.456) (R Core Team 2020; RStudio Team 2020). Data were inspected for normality prior to analysis and transformed to maximize the normality of the model residuals (the exponent used was recorded for each analysis, included in figures or within the analysis tables). For all models, male age, LPS dose (and their interaction), and male relative weight were entered as fixed effects. Where the interaction between LPS dose and age was nonsignificant, we present the model parameters for the interaction, but the remaining model parameters are for models rerun without the interaction. We used relative male weight ([trait value – average trait value]/standard deviation trait value) to control for the decline in weight with male age, as we weighed males just prior to injection (at either 10- or 20-day post adult-eclosion). Tukey’s post-hoc tests were used, where stated, to reveal sources of significance in treatment levels and to control for multiple comparisons.

We used generalized linear models with a binomial error distribution for models exploring the likelihood of: a male dying; male calling or mating and oviposition. For the likelihood of oviposition, female age and weight were included as covariates. We used general linear models to explore variation in the latency to male calling and mating (here, female age, weight and if a second female was required were included as covariates), the number of ejaculates a male produced, and male adult longevity (here, the number of ejaculates produced was included as a covariate). We examined fertility using a generalized linear model with a quasi-binomial error distribution, to control for the significant overdispersion in this model (here, female age and weight were included as covariates).

For analyses of call structure, a principal components analysis (PCA) was used. The PCA returned three axes of variation with eigenvalues greater than one (PC1, PC2, and PC3). These were then used as the response variables in three principal component general linear models.

We analyzed the number of calls in the first calling bout, using a generalized linear model with a Poisson error distribution. We analyzed the total number of calling bouts using a general linear model. We explored variation in the length of individual bouts, the number of calls within the bout and the length of the inter-bout intervals across the 2-min testing period, using general linear models with the addition of bout number as a covariate.

As we have run a considerable number of analyses on our data, we additionally report an *r*^2^ statistic for each model, following Nakagawa (2004).

## RESULTS

### Pre-assay mortality

Of the 90 males assigned to the “young” treatment and 108 males assigned to the “old” treatment, 4.4% and 14.8% died prior to immune challenge, respectively. Of the individuals injected with the control, 0.1%, 0.5%, and 1% LPS solution, 0/47, 1/37, 5/42, and 16/50 died, respectively. The likelihood of dying before the mating assay varied across the LPS treatments (χ^2^ = 27.6; *P* = 0.000004) and decreased with male weight (χ^2^ = 8.69; *P* = 0.003). Post-hoc tests revealed that males who received a 1% dose were more likely to die than males that received any other dose. There was, however, no effect of male age (χ^2^ = 0.51; *P* = 0.48).

We acknowledge the potential for selection due to the increased mortality associated with the 1% LPS dose. Thus, while we have included the 1% LPS dose in our analyses, we also present our analysis with this dose removed in Supplementary Materials (Supplementary Tables S3–S10). However, we note that the results are highly comparable, suggesting a limited effect of this apparent selection on our results.

### Male reproductive investment

#### Male courtship call structure

A PCA was used to summarize variation in calling parameters (following Simmons et al. 2013b). The PCA returned three principal components with an eigenvalue greater than 1 (Supplementary Table S1). These principal components explain 71.1% of the variation in call structure. The variation in call structure was characterized primarily by the first principal component (PC1). Variation in PC1 was associated with chirp duration, trill duration, and chirp pulse number. Principal component 2 (PC2) was associated with chirp pulse duration, chirp-trill interval, and trill pulse interval and trill pulse duration. Principal component 3 (PC3) exhibited strong correlation with chirp pulse interval and trill pulse number. Male courtship call structure (summarized by PC1, PC2, and PC3) was not affected by the experimental treatments, or male body size (Table 1).

**Table 1:** Model summaries for the effect of LPS dose, male age, and their interaction on the three principal components

	PC1 (*R*^2^ = 0.05)			PC2 (*R*^2^ = 0.09)			PC3 (*R*^2^ = 0.03)		
	Parameter estimate	Statistic	*P*	Parameter estimate	Statistic	*P*	Parameter estimate	Statistic	*P*
Intercept	−0.25 ± 0.39	*t* = 0.65	0.52	0.24 ± 0.34	*t* = 0.70	0.49	0.01 ± 0.33	*t* = 0.04	0.97
Age		*F*_1,78_ = 1.04	0.31		*F*_1,78_ = 0.0001	0.90		*F*_1,78_ = 0.09	0.90
Age (young)	0.43 ± 0.42	*t* = 1.02	0.31	−0.00 ± 0.38	*t* = −0.01	0.99	0.03 ± 0.36	*t* = 0.10	0.92
LPS		*F*_3,78_ = 0.38	0.77		*F*_3,78_ = 1.15	0.33		*F*_3,78_ = 0.27	0.85
LPS (0.1)	0.36 ± 0.47	*t* = 0.76	0.45	−0.05 ± 0.42	*t* = −0.13	0.90	0.15 ± 0.40	*t* = 0.38	0.71
LPS (0.5)	−0.08 ± 0.50	*t* = 0.17	0.87	−0.77 ± 0.44	*t* = −1.73	0.09	−0.07 ± 0.42	*t* = −0.18	0.86
LPS (1)	−0.12 ± 0.49	*t* = −0.25	0.80	−0.26 ± 0.44	*t* = −0.59	0.56	−0.23 ± 0.41	*t* = −0.55	0.58
Relative male weight	−0.31 ± 0.21	*F*_3,78_ = 2.13	0.15	0.18 ± 0.19	*F*_1,78_ = 0.91	0.34	0.01 ± 0.18	*F*_1,78_ = 0.001	0.97
LPS × Age		*F*_3,75_ = 0.19	0.90		*F*_3,75_ = 0.99	0.40		*F*_3,75_ = 0.37	0.78
LPS (0.1) × Age (young)		*t* = 0.27	0.79		*t* = 1.14	0.26		*t* = −0.84	0.41
LPS (0.5) × Age (young)		*t* = −0.50	0.62		*t* = 0.94	0.35		*t* = 0.11	0.92
LPS (1) × Age (young)		*t* = 0.14	0.89		*t* = −0.40	0.69		*t* = −0.59	0.56

The term after the ± is standard error. Parameter estimates for each level of non-significant LPS dose × age interactions are not reported.

#### Male calling investment

The likelihood that a male called was unrelated to LPS dose, age, or relative male weight (Table 2). For all sample sizes used in the following models, see Supplementary Table S2.

**Table 2:** The effect of male age and LPS dose on male calling behavior

	Likelihood of calling (*R*^2^ = 0.06)			Latency to calling (^0.04) (*R*^2^ = 0.1)		
	Parameter estimate	Statistic	*P*	Parameter estimate	Statistic	*P*
Intercept	2.03 ± 0.52	*z* = 3.90	**0.00**	1.08 ± 0.006	*t* = 181.02	**0.00**
Age		χ^2^_1_ = 0.14	0.70		*F*_1,95_ = −1.51	0.22
Age (young)	0.16 ± 0.42	*z* = 0.37	0.71	0.007 ± 0.006	*t* = 1.23	0.22
LPS		χ^2^_3_ = 3.31	0.34		*F*_3,95_ = −2.78	0.05
LPS (0.1)	−0.76 ± 0.62	*z* = −1.21	0.23	−0.02 ± 0.008	*t* = −2.14	**0.03**
LPS (0.5)	−0.54 ± 0.64	*z* = −0.85	0.87	0.004 ± 0.008	*t* = 0.53	0.60
LPS (1)	−1.07 ± 0.62	*z* = −1.73	0.08	−0.01 ± 0.008	*t* = −1.35	0.18
Relative male weight	−0.36 ± 0.23	χ^2^_1_ = 2.59	0.11	0.0005 ± 0.003	*F*_1,95_ = 0.03	0.86
LPS × Age		χ^2^_1_ =1.92	0.59		*F*_3,92_ = 0.09	0.96
LPS (0.1) × Age (young)		*z* = 0.70	0.48		*t* = −0.21	0.83
LPS (0.5) × Age (young)		*z* = −0.12	0.90		*t* = −0.53	0.60
LPS (1) × Age (young)		*z* = 0.97	0.33		*t* = −0.15	0.88

The term after the ± is standard error. Bolded values are significant, with *P* < 0.05. Parameter estimates for each level of non-significant LPS dose × age interactions are not reported.

**Figure 2:**
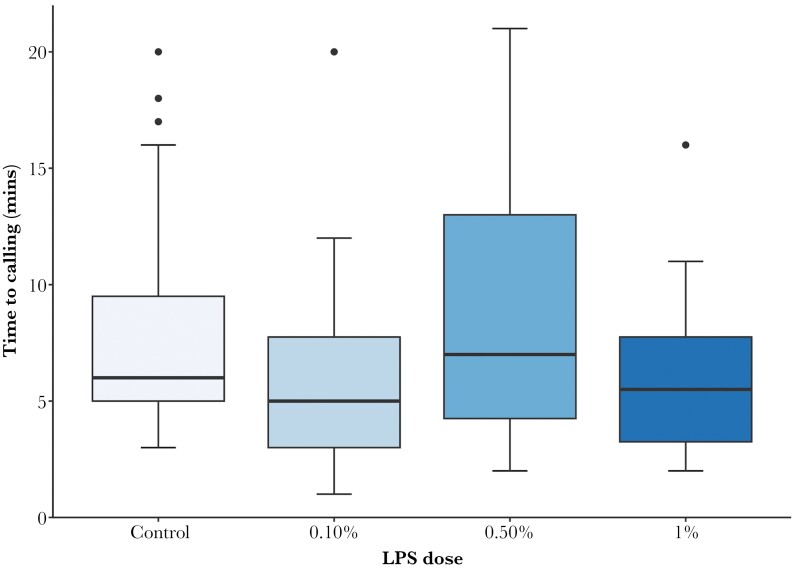
The effect of LPS dose on the latency to calling (mins) (^0.04). The number in parentheses represents the exponent of the transformation. Box plots represent the median (line), interquartile range (box), 95% confidence intervals (whiskers), and outliers (points). *n* Control = 31, 0.1% = 22, 0.5% = 26, 1% = 22.

The latency until a male called varied with the LPS dose (Table 2, Figure 2). Post-hoc tests revealed a difference between the 0.5% and 0.1% LPS dose. Latency until calling was not affected by male age, or relative male weight.

**Figure 3:**
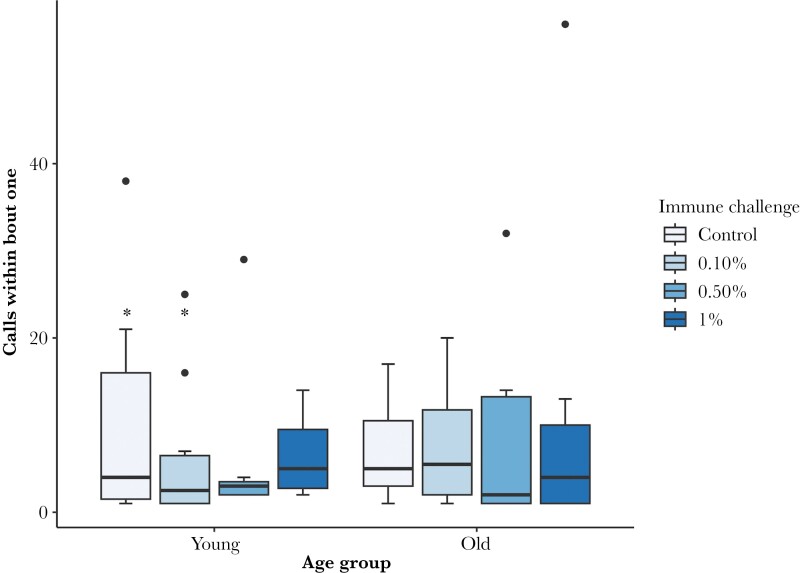
The effect of male age and LPS dose on the number of calls in the first calling bout. Box plots represent the median (line), interquartile range (box), 95% confidence intervals (whiskers) and outliers (points). * indicates a significance of <0.05. *n* young Control = 11, 0.1% = 10, 0.5% = 7, 1% = 8, *n* old Control = 11, 0.1% = 10, 0.5% = 12, 1% = 9.

The number of calls in the first calling bout was affected by an interaction between age and LPS dose (Table 3, Figure 3), as well as male weight. Post hoc tests reveal this was driven by “young” males who received a 0.1% dose calling significantly less than “young” males who received a control solution. We recognize that we have run numerous tests, however, the *R*^2^ value for this model suggests it is a good fit (Table 3).

**Table 3:** The effect of male age and LPS dose on male calling bouts

	Calls within bout one (*R*^2^ = 0.34)			No. of calling bouts (^0.6) (*R*^2^ = 0.11)			Bout length (^0.4) (*R*^2^ = 0.03)		
	Parameter estimate	Statistic	*P*	Parameter estimate	Statistic	*P*	Parameter estimate (×10^−2^)	Statistic	*P*
Intercept	1.94 ± 0.12	*t* = 16.66	**0.00**	1.51 ± 0.12	*t* = 12.68	**0.00**	112 ± 0.90	*t* = 121.41	**0.00**
Age		χ^2^_1_ = 5.82	**0.02**		*F*_1,178_ = 1.02	0.98		*F*_1,178_ = 0.004	0.95
Age (young)	0.36 ± 0.15	*t* = 2.39	**0.02**	0.37 ± 0.16	*t* = 2.31	**0.02**	−0.04 ± 0.70	*t* = −0.06	0.95
LPS		χ^2^_3_ = 6.68	0.08		*F*_3,178_ = 1.02	0.31		*F*_3,178_ = 0.27	0.85
LPS (0.1)	0.12 ± 0.16	*t* = 0.71	0.48	0.49 ± 0.15	*t* = 3.20	**0.002**	−0.60 ± 0.90	*t* = −0.62	0.54
LPS (0.5)	0.005 ± 0.16	*t* = 0.03	0.98	0.38 ± 0.15	*t* = 2.49	**0.01**	−0.70 ± 0.90	*t* = −0.75	0.45
LPS (1)	0.35 ± 0.16	*t* = 2.20	**0.03**	0.71 ± 0.16	*t* = 4.42	**<0.001**	−0.8 ± 0.1	*t* = −0.80	0.42
Relative male weight	−0.15 ± 0.05	χ^2^_1_ = 10.82	**0.001**	0.07 ± 0.04	*F*_1,178_= 2.51	0.11	−0.1 ± 0.4	*F*_1,178_ = 0.13	0.72
Bout number	NA	NA		NA	NA		−0.4 ± 0.3	*F*_1,178_ = 1.94	0.17
LPS × Age		χ^2^_3_ = 13.24	**0.004**		*F*_3,175_ = 3.80	**0.01**		*F*_3,17 5_= 0.52	0.67
LPS (0.1) × Age (young)	−0.62 ± 0.23	*t* = −2.74	**0.006**	−0.52 ± 0.22	*t* = −2.40	**0.02**		*t* = −0.66	0.51
LPS (0.5) × Age (young)	−0.48 ± 0.24	*t* = −2.02	**0.04**	−0.36 ± 0.23	*t* = −1.59	0.11		*t* = 0.54	0.59
LPS (1) × Age (young)	−0.76 ± 0.23	*t* = −3.28	**0.001**	−0.74 ± 0.23	*t* = −3.24	**0.001**		*t* = −0.25	0.81

The term after the ± is standard error. Bolded values are significant, with *P* < 0.05. Parameter estimates for each level of non-significant LPS dose × age interactions are not reported.

Similarly, we also examined the length of each bout, and the number of calls within each bout. Male age, LPS dose and male weight had no effect on these traits (Tables 3 and 4). However, the inter-bout interval was longer for older males, and increased with bout number, but was not affected by LPS dose or male weight (Table 4). Moreover, male age and LPS dose had an interactive effect on the total number of calling bouts (Table 3). Post-hoc tests revealed that older males, but not younger males, who received a 0.1% or 1% dose of LPS had more calling bouts than older control males.

**Table 4:** The effect of male age and LPS dose on male calling bouts (continued)

	Calls within each bout (^0.04) (*R*^2^ = 0.03)			Inter-bout interval (^0.04) (*R*^2^ = 0.12)		
	Parameter estimate	Statistic	*P*	Parameter estimate	Statistic	*P*
Intercept	1.06 ± 0.008	*t* = 130.12	**0.00**	1.13 ± 0.01	*t* = 127.75	**0.00**
Age		*F*_1,178_ = 0.002	0.97		*F*_1,100_ = 4.01	**0.05**
Age (young)	−0.0002 ± 0.006	*t* = −0.04	0.97	−0.01 ± 0.01	*t* = −2.00	**0.05**
LPS		*F*_3,178_ = 0.48	0.70		*F*_3,100_ = 0.77	0.51
LPS (0.1)	−0.009 ± 0.008	*t* = −1.07	0.29		*t* = −1.46	0.15
LPS (0.5)	−0.004 ± 0.008	*t* = −0.54	0.60	−0.01 ± 0.01	*t* = −0.58	0.56
LPS (1)	−0.001 ± 0.009	*t* = −0.12	0.90	−0.01 ± 0.01	*t* = −0.89	0.38
Relative male weight	0.0002 ± 0.003	*F*_1,178_ = 0.003	0.97	−0.004 ± 0.003	*F*_1,100_ = 1.70	0.20
Bout number	−0.004 ± 0.002	*F*_1,178_ = 3.09	0.08	−0.01 ± 0.002	*F*_1,100_ = 4.75	**0.03**
LPS × Age		*F*_3,175_ = 0.04	0.99		*F*_3,97_ = 0.41	0.74
LPS (0.1) × Age (young)		*t* = −0.17	0.87		*t* = −0.15	0.88
LPS (0.5) × Age (young)		*t* = 0.15	0.88		*t* = 0.003	0.99
LPS (1) × Age (young)		*t* = −0.14	0.89		*t* = 0.79	0.43

The term after the ± is standard error. Bolded values are significant, with *P* < 0.05. Parameter estimates for each level of non-significant LPS dose × age interactions are not reported.

We also examined variation in male mating behavior (the latency to mating, the likelihood of mating success, and total number of matings achieved). We found no impact of LPS dose, male age (or their interaction), or male weight on these traits (Table 5).

**Table 5:** The effect of male age and LPS dose on male mating behavior

	Likelihood of mating (*R*^2^ = 0.02)			Latency to mating (^0.52) (*R*^2^ = 0.18)			Number of ejaculates transferred (^1.04) (*R*^2^ = 0.04)		
	Parameter estimate	Statistic	*P*	Parameter estimate	Statistic	*P*	Parameter estimate	Statistic	*P*
Intercept	−0.23 ± 0.35	z = −0.66	0.51	3.22 ± 1.24	*t* = 2.60	**0.01**	1.45 ± 0.12	*t* = 12.06	**0.00**
Age		χ^2^_1_ = 0.00003	0.99		*F*_1,72_ = 1.56	0.22		*F*_1,147_ = 0.92	0.33
Age (young)	−0.002 ± 0.34	z = −0.005	0.99	0.26 ± 0.21	*t* = 1.30	0.22	−0.11 ± 0.12	*t* = −0.96	0.34
LPS		χ^2^_3_ = 1.59	0.66		*F*_3,72_ = 0.69	0.92		*F*_3,147_ = 1.13	0.34
LPS (0.1)	−0.32 ± 0.46	z =−0.69	0.49	−0.22 ± 0.29	*t* = −0.77	0.45	−0.02 ± 0.16	*t* = −0.15	0.88
LPS (0.5)	−0.48 ± 0.46	z =−1.04	0.30	−0.25 ± 0.28	*t* = −0.91	0.37	−0.005 ± 0.16	*t* = −0.03	0.98
LPS (1)	−0.51 ± 0.48	z =−1.08	0.28	0.14 ± 0.30	*t* = 0.47	0.64	−0.26 ± 0.16	*t* = −1.64	0.10
Relative male weight	−0.12 ± 0.18	χ^2^_1_ = 0.43	0.75	−0.01 ± 0.11	*F*_1,72_ = 0.01	0.92	−0.03 ± 0.06	*F*_1,147_ = 0.23	0.63
Female weight	NA	NA		−0.55 ± 1.06	*F*_1,72_ = 0.27	0.92	NA	NA	
Female age	NA	NA		−0.003 ± 0.10	*F*_1,72_ = 0.009	0.98	NA	NA	
Second female (0/1)	NA	NA		−0.69 ± 0.23	*F*_1,72_ = 9.07	**0.004**	NA	NA	
LPS × Age		χ^2^_3_ = 1.19	0.75		*F*_3,72_ = 0.27	0.85		*F*_3,144_ = 0.68	0.57
LPS (0.1) × Age (young)		z =−0.52	0.60		*t* = −0.38	0.71		*t* = −0.22	0.83
LPS (0.5) × Age (young)		z =0.35	0.73		*t* = −0.57	0.57		*t* = 1.04	0.30
LPS (1) × Age (young)		z =0.58	0.57		*t* = 0.35	0.72		*t* = 0.81	0.42

The term after the ± is standard error. Bolded values are significant, with *P* < 0.05. Parameter estimates for each level of non-significant LPS dose × age interactions are not reported.

#### Male offspring production

The likelihood of a male’s partner laying eggs was affected by LPS dose (Table 6). Post hoc tests reveal this was driven by males who received a 1% dose siring more offspring than males who received a 0.1% dose of LPS. The likelihood of laying eggs increased with female weight, but decreased with male weight and was not affected by male or female age.

**Table 6:** The effect of male age, LPS dose and their interaction on female reproductive output

	Likelihood of laying eggs (*R*^2^ = 0.24)			Number of eggs laid (^0.84) (*R*^2^ = 0.13)			Fertility (proportion of eggs hatched) (*R*^2^ = 0.35)		
	Parameter estimate	Statistic	*P*	Parameter estimate	Statistic	*P*	Parameter estimate	Statistic	*P*
Intercept	−5.09 ± 3.35	z = −1.52	0.13	127.43 ± 57.27	*t* = 2.23	**0.03**	−5.13 ± 3.68	z = −1.39	0.16
Age		χ^2^_1_ = 0.35	0.56		*F*_1,31_ = 0.001	0.97		χ^2^_1_ = 3.09	0.38
Age (young)	−0.31 ± 0.54	z = −0.59	0.56	2.79 ± 9.82	*t* = 0.28	0.78	−0.63 ± 0.62	z = −1.01	0.31
LPS		χ^2^_3_ = 9.78	**0.02**		*F*_3,31_ = 0.21	0.88		χ^2^_3_ = 1.02	0.31
LPS (0.1)	−1.40 ± 0.71	z = −1.96	**0.05**	−4.12 ± 14.17	*t* = −0.29	0.77	1.12 ± 0.86	z = 1.30	0.20
LPS (0.5)	−0.60 ± 0.69	z = −0.87	0.38	−1.89 ± 13.40	*t* = −0.14	0.89	1.15 ± 0.74	z = 1.55	0.12
LPS (1)	1.34 ± 0.92	z = −1.45	0.14	−3.60 ± 12.04	*t* = −0.30	0.70	0.55 ± 0.75	z = 0.75	0.46
Relative male weight	−0.64 ± 0.28	χ^2^_1_ = 5.71	**0.02**	8.06 ± 5.53	*F*_1,31_ = 2.32	0.14	0.25 ± 0.34	χ^2^_1_ = 0.54	0.46
Female weight	6.56 ± 2.76	χ^2^_1_ = 6.56	**0.01**	−50.23 ± 47.29	*F*_1,31_ = 0.54	0.47	−0.73 ± 2.94	χ^2^_1_ = 0.06	0.80
Female age	0.20 ± 0.25	χ^2^_1_ = 0.65	0.42	−4.46 ± 4.15	*F*_1,31_ = 0.53	0.47	0.38 ± 0.27	χ^2^_1_ = 1.88	0.17
Age × LPS		χ^2^_3_ = 2.95	0.40		*F*_3,28_ = 0.47	0.70		χ^2^_3_ = 5.43	0.14
LPS (0.1) × Age (young)		z = −0.08	0.93		*t* = 1.14	0.26		z = 1.67	0.10
LPS (0.5) × Age (young)		z = 0.76	0.45		*t* = 0.53	0.60		z = −1.00	0.32
LPS (1) × Age (young)		z = −0.01	0.99		*t* = 0.10	0.92		z = −0.29	0.77

The term after the ± is standard error. Bolded values are significant, with *P* < 0.05. Parameter estimates for each level of non-significant LPS dose × age interactions are not reported.

We also examined variation in the total number of eggs sired by a male, and the proportion that hatched. Neither of these traits were affected by LPS dose, male age, male weight, or female age or weight (Table 6).

#### Spermatophore weight

The weight of the spermatophore produced by a male was positively related to male age and relative male weight, but was unaffected by LPS dose, or the number of the male’s prior matings (Table 7).

**Table 7:** The effect of male age, LPS dose and their interaction on the weight of spermatophore and male longevity

	Weight of spermatophore (*R*^2^ = 0.27)			Male longevity (^0.04) (*R*^2^ = 0.17)		
	Parameter estimates (×10^−4^)	Statistic	*P*	Parameter estimates	Statistic	*P*
Intercept	20.0 ± 0.6	*t* = 28.35	**0.00**	1.15 ± 0.005	*t* = 249.84	**0.00**
Age		*F*_1,103_ = 14.90	**0.0002**		*F*_1,146_ = 12.30	**<0.0001**
Age (young)	−2.0 ± 0.5	*t* = −3.85	**0.0002**	−0.01 ± 0.003	*t* = 4.04	**<0.0001**
LPS		*F*_3,103_ = 1.10	0.35		*F*_3,146_ = 1.07	0.36
LPS (0.1)	0.6 ± 0.7	*t* = 0.85	0.40	−0.005 ± 0.004	*t* = −1.26	0.21
LPS (0.5)	−0.6 ± 0.6	*t* = −0.97	0.33	−0.002 ± 0.004	*t* = −0.57	0.57
LPS (1)	−0.3 ± 0.7	*t* = −0.38	0.71	−0.007 ± 0.004	*t* = −1.65	0.10
Relative male weight	0.9 ± 0.3	*F*_1,103_ = 13.19	**0.0004**	0.002 ± 0.001	*F*_1,146_ = 2.35	0.3
Number of prior matings	0.07 ± 0.5	*F*_1,103_ = 0.02	0.89	0.004 ± 0.002	*F*_1,146_ = 2.63	0.11
Age × LPS		*F*_1,100_ = 1.26	0.29		*F*_1,146_ = 1.25	0.29
LPS (0.1) × Age (young)		*t* = −0.39	0.70		*t* = −1.33	0.19
LPS (0.5) × Age (young)		*t* = −1.37	0.17		*t* = 0.06	0.95
LPS (1) × Age (young)		*t* = 1.71	0.09		*t* = 0.69	0.49

The term after the ± is standard error. Bolded values are significant, with *P* < 0.05. Parameter estimates for each level of non-significant LPS dose × age interactions are not reported.

### Male adult longevity

Adult longevity was not affected by LPS dose, the number of previous matings or male weight. However, longevity post-injection was affected by male age, with males assigned to the older treatment group living longer than those assigned to the younger group (Table 7).

## DISCUSSION

We explored the impact of male RRV on terminal investment strategies, to test specifically for evidence of a dynamic terminal investment threshold. Our data revealed three key findings related to terminal investment in *T. oceanicus*. First, it provides limited support for the dynamic terminal investment threshold: there was no consistent evidence of a positive interaction between male age and immune challenge intensity on male reproductive investment. Second, we found some evidence of terminal investment; older males produced a larger spermatophore than younger males. Third, male calling rate was age-dependent: older males had a slower calling rate (longer inter-bout intervals), compared with younger males. The increased ejaculate size combined with a reduced calling intensity in older males may reflect a condition-dependent trade-off between pre- and post-copulatory traits in *T. oceanicus*.

Evidence for the dynamic terminal investment threshold should demonstrate a significant interaction between intrinsic and/or extrinsic cues indicating a reduction in RRV. We predicted a significant interaction between LPS dose and male age, where even at low doses of an immune challenge, older individuals should display terminal investment via upregulated reproductive effort. In contrast, for younger individuals, we hypothesized that only higher doses of LPS would elicit terminal investment. However, we found no clear evidence that either of these scenarios held for any of the reproductive traits measured. Although, we note that our relatively small sample sizes mean that we have low statistical power. We observed two interactions between LPS dose and age. First, in the number of calls produced by a male in his first calling bout: young males who received a 0.1% dose of LPS produced fewer calls in their first bout than males who received a control solution. While for older males there was no difference in calls for the different LPS doses. This result provides tentative evidence of a dynamic terminal investment threshold, whereby younger males show a cost of resistance at low doses of an immune challenge, but then revert back into investment at higher doses, while older males maintain reproductive effort. However, this failed to meet our criteria for terminal investment, as the average call rate of the immune challenged males did not exceed that of the control. Rather, as courtship calls are critical to secure a mating in this species, this suggests that young males may downregulate reproductive investment (Copeland and Fedorka 2012; Duffield et al. 2018), potentially to increase immune response against the immune challenge. Why this should occur for young but not old males is not clear. Such fundamental trade-offs between reproduction and immunity are widespread in both invertebrates (Lawniczak et al. 2007; Brokordt et al. 2019) and vertebrates (Judson et al. 2020). Indeed, a similar reproductive trade-off was demonstrated in *T. oceanicus*, in which males reduced their sperm viability in response to a 0.1% dose of LPS (Simmons 2012). However, while dose-dependent effects of terminal investment are common (Schwanz 2008; Duffield et al. 2018), the relationship is not always linear (Kivleniece et al. 2010; Krams et al. 2011; Hendry et al. 2016). This raises the possibility that an alternative physiological process, such as hormesis, may be occurring in conjunction with aspects of terminal investment. Hormesis is a phenomenon where individuals respond strongly to a low than a high dose, of a biological stressor (Calabrese and Mattson 2017), which could explain the reduction in reproduction at low doses of an immune elicitor and a return to normal reproduction at a higher dose, as seen in terminal investment studies. Disentangling these processes requires further investigation. However, defining terminal investment as an increase in reproduction that *exceeds* the control level, should reduce the potential for it to be confounded with dose-dependent physiological processes, such as hormesis.

Interestingly, our second interactive effect between age and LPS dose was in the total number of calling bouts in the 2-min recording window. Older males given a 0.1% or a 1% (but not 0.5%) dose of LPS had more calling bouts than older control males. This is in contrast to our earlier result demonstrating that young males who received a 0.1% dose of LPS produced fewer calls in their first bout than young males who received a control solution. This suggests that investment into calling is not temporally stable. Moreover, as this pattern did not extend to older males given a 0.5% dose of LPS it is unclear if this result is clear evidence of terminal investment.

We did not find clear evidence for the dynamic terminal investment threshold, but consistent with the traditional terminal investment hypothesis, older males (who have intrinsically lower RRVs) produced larger spermatophores. Increased male ejaculate size often correlates with greater male reproductive success, particularly in highly polyandrous species, such as *T. oceanicus* (Wedell 1997; Simmons 2001). In Orthoptera, the weight of a spermatophore is indicative of not only the amount of sperm inside (Sturm 2014), but also the quantity of seminal proteins, which also affect fertility (Simmons et al. 2013a). Larger quantities of these non-sperm components provide a significant competitive advantage, improving male reproductive success by improving both sperm (Wigby et al. 2009; Simmons and Beveridge 2011) and offspring viability (Fricke et al. 2009; Simmons et al. 2013a).

However, an increase in spermatophore weight is only biologically significant if it increases male fitness. We found no evidence that increased spermatophore weight correlated with the older male’s reproductive success: neither male age nor spermatophore weight affected female fecundity. There are three potential explanations for this apparent absence of a fitness effect. First, female reproductive output was collected over just eight days (approximately 55% of the female reproductive window) (N.R., personal observation). Thus, our sampling methodology may have reduced the likelihood of detecting a fitness advantage for increased male ejaculate size. Second, changes in spermatophore weight may have affected unmeasured fitness components. For example, seminal fluid proteins can cause physiological and behavioral post-mating changes in females (Gillott 2003; Avila et al. 2011), such as the induction of a refractory period (Liu and Kubli 2003; Fricke et al. 2009). Similarly, apolipophorin-III, an important mediator of antibacterial immunity in crickets (Adamo et al. 2008), which is found in the seminal fluid of *T. oceanicus* may protect females from bacteria introduced into their reproductive tract during mating (Simmons et al. 2013a). An assessment of changes in female post-mating receptivity, longevity and immunity, all of which may indirectly affect male reproductive success, may provide insight into whether the larger spermatophores of older males confers a yet unmeasured fitness advantage. Finally, males with larger spermatophores, and thus seminal fluid volume, may be better sperm competitors (Simmons and Beveridge 2011). While we only allowed a single mating, *T. oceanicus* females are highly polyandrous (Tregenza and Wedell 1998; Simmons 2001), and a fitness benefit for larger spermatophores may only manifest in multiply mated females.

Male RRV had a mixed impact on the reproductive output of their partners. Females who mated with males that received a 1% LPS dose had a higher likelihood of laying eggs than males that received a 0.1% dose. However, as males at the highest dose did not exceed control males in their likelihood of siring offspring, this is not compelling evidence of terminal investment. Furthermore, females who mated with males challenged with a 1% LPS dose did not demonstrate a greater fecundity or fertility. Therefore, despite a higher likelihood of egg laying, the 1% LPS dose does not appear to be having a direct impact on male fitness (e.g., via greater offspring production). This could be due to male ejaculate quality. In *T. oceanicus,* sperm viability, the proportion of live sperm in ejaculate, is a key predictor of male fertilization success (Garcia-Gonzalez and Simmons 2005). However, a trade-off exists between sperm viability and immunity, where *T. oceanicus* males with increased immunocompetence have reduced sperm viability (Simmons and Roberts 2005; Simmons 2012). A future terminal investment study focusing on sperm viability and seminal fluid proteins could provide key information on the trade-offs between reproduction and immunity.

Interestingly, aside from increased spermatophore weight, there was no other evidence of terminal investment in older males, despite male age eliciting terminal investment behaviors in a number of pre- (Copeland and Fedorka 2012; Gonzalez-Tokman et al. 2013; Duffield et al. 2018) and post-copulatory traits in other species (Velando et al. 2006; Win et al. 2013; Farchmin et al. 2020). Rather, we found that older males had a slower calling rate (i.e., longer inter-bout intervals) for their courtship song. Female *T. oceanicu*s, prefer calls with a higher duty cycle (more sound per unit time) (Rebar et al. 2009), and in the sister species *T. commodus*, females show a strong preference for male calls which are produced at a higher rate (Bentsen et al. 2006; Drayton et al. 2012). There are two potential reasons why older males had a decline in calling rate. First, it may reflect senescence, due to decreased metabolic rate (Hack 1997) or the deterioration of muscles associated with the forewings (Sohal 1976). Second, the energetically expensive calling rate may be traded-off against increased ejaculate weight as males age. Sperm competition theory suggests a trade-off between investment in attracting a mate and investment in ejaculate quality (Parker 1998; Parker et al. 2013). Therefore, older males, may prioritize investment in ejaculate quality over courtship call quality (which is required to elicit a copulation from an already-present female). Indeed, there is evidence of a trade-off between the courtship call and the ejaculate of *T. oceanicus* (Simmons et al. 2010), where increased sperm viability is associated with a reduction in the trill element of the courtship call (females prefer calls with longer trill elements; Simmons et al. 2013b). Although we did not find any differences in the microstructure of *T. oceanicus* calls in response to age or LPS dose, it is possible that the costly calling rate is being traded-off against spermatophore investment.

There are several issues surrounding the use of LPS as an immune elicitor. LPS can be cytotoxic and can often be contaminated with peptidoglycan (derived from the cell wall of gram-positive bacteria) (Tanaka et al. 2009) and this may have impacted mortality. We also note that because of its toxicity, a reduced RRV in higher doses of LPS may not just reflect a simulated immune infection, but a response to toxicity. Indeed, it may be that the higher doses caused extreme sickness in males, to the point where they could not increase their investment, resulting in a lack of terminal investment. Moreover, studies suggest that peptidoglycan, rather than LPS is largely responsible for the stimulation of the immune response in insects (Leulier et al. 2003; Kaneko et al. 2004). Nevertheless, the widespread use of LPS as an immune elicitor (Adamo 1999; Jacot et al. 2004; Reaney and Knell 2010; Bowers et al. 2012), and its ability to elicit an immune response in *T. oceanicus* (Simmons 2012), suggests it is an appropriate tool to stimulate immune investment and manipulate an individual’s RRV.

Our study also demonstrates the importance of considering selection when examining terminal investment: our experiment was subject to potential selection at two points. First, as approximately 15% of males allocated to the “old” group died before their immune challenge treatment, there may have been selection for high quality males in this cohort. While this is supported by the greater longevity of “old” males, there was, however, no evidence of reduced variance (indicative of selection) in any of the fitness traits measured for older males. Second, males that survived the 1% LPS dose (LD_30_: where the dosage killed 30% of the population), should, theoretically, be of higher quality than males in the other cohorts, as immunocompetence is a condition-dependent trait (Moller and Petrie 2002). Other studies have also used high doses of bacteria to challenge individuals (e.g., LD_27_) (Adamo 1999; Shoemaker et al. 2006), but did not consider the impact of selection in their interpretation. Ultimately, however, selection did not appear to play a significant role in our study as there was no clear evidence of reduced variance in fitness traits in either the older male cohort, or in the higher LPS dose treatments. Moreover, we analyzed our results both with and without the highest LPS dose (in which selection was strongest), and they were qualitatively the same, except for one trait (the likelihood of oviposition). While selection did not appear to play a major role in the reproductive traits that we analyzed, the potential for selection should be formally considered in studies exploring terminal investment.

In conclusion, we found only limited support for the dynamic terminal investment threshold: there was no clear evidence of a consistent positive interaction between male age and immune challenge intensity in 16 of 18 traits investigated. However, we found evidence for age-related terminal investment: older males increased their ejaculate volume compared with younger males, but also had a slower calling rate. This suggests a potential trade-off between these two pre- and post-copulatory traits. While *T. oceanicus* females prefer higher calling rates, increased ejaculate size is likely to be important in determining male competitive fertilization success in this highly polyandrous species, potentially prioritizing increased post-copulatory investment as a terminal investment strategy.

## Supplementary Material

arad021_suppl_Supplementary_Material

## Data Availability

Analyses reported in this article can be reproduced using the data provided by Rutkowski et al (2023).

## References

[CIT0001] Adamo SA. 1999. Evidence for adaptive changes in egg laying in crickets exposed to bacteria and parasites. Anim Behav. 57:117–124. 10053078 10.1006/anbe.1998.0999

[CIT0002] Adamo SA , Jensen M, Younger M. 2001. Changes in lifetime immunocompetence in male and female *Gryllus texensis* (formerly *G-integer*): trade-offs between immunity and reproduction. Anim Behav. 62:417–425.

[CIT0003] Adamo SA , Roberts JL, Easy RH, Ross NW. 2008. Competition between immune function and lipid transport for the protein apolipophorin III leads to stress-induced immunosuppression in crickets. J Exp Biol. 211(4):531–538. 18245629 10.1242/jeb.013136

[CIT0004] Audacity Team. 2021. Audacity(R): Free Audio Editor and Recorder [Computer application]. Version 3.0.0. https://audacityteam.org/

[CIT0005] Avila FW , Sirot LK, Laflamme BA, Rubinstein CD, Wolfner MF. (2011). Insect seminal fluid proteins: identification and function. In: M. R. Berenbaum, R. T. Carde and G. E. Robinson editors. Annual Review of Entomology, Vol 56. p. 21–40. 10.1146/annurev-ento-120709-144823 PMC3925971 20868282

[CIT0006] Barribeau SM , Sok D, Gerardo NM. 2010. Aphid reproductive investment in response to mortality risks. BMC Evol Biol. 10:251. 20716370 10.1186/1471-2148-10-251 PMC2940815

[CIT0007] Bentsen CL , Hunt J, Jennions MD, Brooks R. 2006. Complex multivariate sexual selection on male acoustic signaling in a wild population of *Teleogryllus commodus*. Am Nat. 167(4):E102–E116. 16670989 10.1086/501376

[CIT0008] Bonneaud C , Mazuc J, Chastel O, Westerdahl H, Sorci G. 2004. Terminal investment induced by immune challenge and fitness traits associated with major histocompatibility complex in the house sparrow. Evolution. 58(12):2823–2830. 15696759 10.1111/j.0014-3820.2004.tb01633.x

[CIT0009] Bowers EK , Smith RA, Hodges CJ, Zimmerman LM, Thompson CF, Sakaluk SK. 2012. Sex-biased terminal investment in offspring induced by maternal immune challenge in the house wren (*Troglodytes aedon*). Proceedings of the Royal Society B-Biological Sciences. 279(1739):2891–2898. 10.1098/rspb.2012.0443 PMC3367793 22456887

[CIT0010] Brokordt K , Defranchi Y, Esposito I, Carcamo C, Schmitt P, Mercado L, De La Fuente-Ortega E, Rivera-Ingraham GA. 2019. Reproduction immunity trade-off in a *Mollusk*: hemocyte energy metabolism underlies cellular and molecular immune responses. Front Physiol. 10:1–16. 30804806 10.3389/fphys.2019.00077 PMC6378683

[CIT0011] Calabrese EJ , Mattson MP. 2017. How does hormesis impact biology, toxicology, and medicine? NPJ Aging Mech Dis. 3:1–8. 28944077 10.1038/s41514-017-0013-z PMC5601424

[CIT0012] Clutton-Brock TH. 1984. Reproductive effort and terminal investment in iteroparous animals. Am Nat. 123(2):212–229.

[CIT0013] Copeland EK , Fedorka KM. 2012. The influence of male age and simulated pathogenic infection on producing a dishonest sexual signal. Proceedings of the Royal Society B-Biological Sciences. 279(1748):4740–4746. 10.1098/rspb.2012.1914 PMC3497096 23034704

[CIT0014] Cotter SC , Ward RJS, Kilner RM. 2011. Age-specific reproductive investment in female burying beetles: independent effects of state and risk of death. Funct Ecol. 25(3):652–660.

[CIT0015] Curio E. 1983. Why do young birds reproduce less well? Ibis. 125(3):400–404.

[CIT0016] Descamps S , Boutin S, Berteaux D, Gaillard JM. 2007. Female red squirrels fit Williams’ hypothesis of increasing reproductive effort with increasing age. J Anim Ecol. 76(6):1192–1201. 17922715 10.1111/j.1365-2656.2007.01301.x

[CIT0017] Desrochers A. 1992. Age and foraging success in European blackbirds - variation between and within individuals. Anim Behav. 43(6):885–894.

[CIT0018] Drayton JM , Hall MD, Hunt J, Jennions MD. 2012. Sexual signaling and immune function in the black field cricket *Teleogryllus commodus*. PLoS One. 7(7):e39631. 22808047 10.1371/journal.pone.0039631 PMC3392257

[CIT0019] Duffield KR , Bowers EK, Sakaluk SK, Sadd BM. 2017. A dynamic threshold model for terminal investment. Behav Ecol Sociobiol. 71(12):1–13. 10.1007/s00265-017-2416-z PMC6039117 30002566

[CIT0020] Duffield KR , Hampton KJ, Houslay TM, Hunt J, Rapkin J, Sakaluk SK, Sadd BM. 2018. Age-dependent variation in the terminal investment threshold in male crickets. Evolution. 72(3):578–589. 29392709 10.1111/evo.13443

[CIT0021] Duffield KR , Hampton KJ, Houslay TM, Hunt J, Sadd B, Sakaluk SK. 2019. Inbreeding alters context-dependent reproductive effort and immunity in male crickets. J Evol Biol. 32(7):731–741. 30985046 10.1111/jeb.13478 PMC13741172

[CIT0022] Duffield KR , Hampton KJ, Houslay TM, Rapkin J, Hunt J, Sadd BM, Sakaluk SK. 2020. Macronutrient intake and simulated infection threat independently affect life history traits of male decorated crickets. Ecol Evol. 10(20):11766–11778. 33144999 10.1002/ece3.6813 PMC7593159

[CIT0023] Farchmin PA , Eggert AK, Duffield KR, Sakaluk SK. 2020. Dynamic terminal investment in male burying beetles. Anim Behav. 163:1–7.

[CIT0024] Fricke C , Wigby S, Hobbs R, Chapman T. 2009. The benefits of male ejaculate sex peptide transfer in *Drosophila melanogaster*. J Evol Biol. 22(2):275–286. 19032499 10.1111/j.1420-9101.2008.01638.x

[CIT0025] Froy H , Lewis S, Catry P, Bishop CM, Forster IP, Fukuda A, Higuchi H, Phalan B, Xavier JC, Nussey DH, et al. 2015. Age-related variation in foraging behaviour in the wandering albatross at south georgia: no evidence for senescence. PLoS One. 10(1):e0116415. 25574995 10.1371/journal.pone.0116415 PMC4289070

[CIT0026] Garcia-Gonzalez F , Simmons LW. 2005. Sperm viability matters in insect sperm competition. Curr Biol. 15(3):271–275. 15694313 10.1016/j.cub.2005.01.032

[CIT0027] Ghalambor CK , Martin TE. 2001. Fecundity-survival trade-offs and parental risk-taking in birds. Science. 292(5516):494–497. 11313493 10.1126/science.1059379

[CIT0028] Gillott C. 2003. Male accessory gland secretions: modulators of female reproductive physiology and behavior. Annu Rev Entomol. 48:163–184. 12208817 10.1146/annurev.ento.48.091801.112657

[CIT0029] Gonzalez-Tokman DM , Gonzalez-Santoyo I, Cordoba-Aguilar A. 2013. Mating success and energetic condition effects driven by terminal investment in territorial males of a short-lived invertebrate. Funct Ecol. 27(3):739–747.

[CIT0030] Hack MA. 1997. The effects of mass and age on standard metabolic rate in house crickets. Physiol Entomol. 22(4):325–331.

[CIT0031] Heinze J , Schrempf A. 2012. Terminal investment: individual reproduction of ant queens increases with age. PLoS One. 7(4):e35201. 22509399 10.1371/journal.pone.0035201 PMC3324418

[CIT0032] Hendry TA , Clark KJ, Baltrus DA. 2016. A highly infective plant-associated bacterium influences reproductive rates in pea aphids. R Soc Open Sci. 3(2):150478. 26998321 10.1098/rsos.150478 PMC4785972

[CIT0033] Jacot A , Scheuber H, Brinkhof MWG. 2004. Costs of an induced immune response on sexual display and longevity in field crickets. Evolution 58(10):2280–2286. 15562690 10.1111/j.0014-3820.2004.tb01603.x

[CIT0034] Jehan C , Sabarly C, Rigaud T, Moret Y. 2021. Late-life reproduction in an insect: Terminal investment, reproductive restraint or senescence. J Anim Ecol. 90(1):282–297. 33051872 10.1111/1365-2656.13367

[CIT0035] Jones TM , Durrant J, Michaelides EB, Green MP. 2015. Melatonin: a possible link between the presence of artificial light at night and reductions in biological fitness. Philos Trans R Soc B Biol Sci 370(1667):1–8. 10.1098/rstb.2014.0122 PMC4375363 25780235

[CIT0036] Judson JM , Reding DM, Bronikowski AM. 2020. Immunosenescence and its influence on reproduction in a long-lived vertebrate. J Exp Biol. 223(12):1–9. 10.1242/jeb.223057 PMC7328165 32376708

[CIT0037] Kaneko T , Goldman WE, Mellroth P, Steiner H, Fukase K, Kusumoto S, Harley W, Fox A, Golenbock D, Silverman N. 2004. Monomeric and polymeric gram-negative peptidoglycan but not purified LPS stimulate the *Drosophila* IMD pathway. Immunity. 20(5):637–649. 15142531 10.1016/s1074-7613(04)00104-9

[CIT0038] Kivleniece I , Krams I, Daukste J, Krama T, Rantala MJ. 2010. Sexual attractiveness of immune-challenged male mealworm beetles suggests terminal investment in reproduction. Anim Behav. 80(6):1015–1021.

[CIT0039] Krams I , Daukste J, Kivleniece I, Krama T, Rantala MJ, Ramey G, Sausa L. 2011. Female choice reveals terminal investment in male mealworm beetles, *Tenebrio molitor*, after a repeated activation of the immune system. J Insect Sci. 11:1–10. 21864151 10.1673/031.011.5601 PMC3281432

[CIT0040] Lafaille M , Bimbard G, Greenfield MD. 2010. Risk trading in mating behavior: forgoing anti-predator responses reduces the likelihood of missing terminal mating opportunities. Behav Ecol Sociobiol. 64(9):1485–1494.

[CIT0041] Lawniczak MKN , Barnes AI, Linklater JR, Boone JM, Wigby S, Chapman T. 2007. Mating and immunity in invertebrates. Trends Ecol Evol 22(1):48–55. 17028056 10.1016/j.tree.2006.09.012

[CIT0042] Leulier F , Parquet C, Pili-Floury S, Ryu JH, Caroff M, Lee WJ, Mengin-Lecreulx D, Lemaitre B. 2003. The *Drosophila* immune system detects bacteria through specific peptidoglycan recognition. Nat Immunol. 4(5):478–484. 12692550 10.1038/ni922

[CIT0043] Liu HF , Kubli E. 2003. Sex-peptide is the molecular basis of the sperm effect in *Drosophila melanogaster*. Proc Natl Acad Sci USA. 100(17):9929–9933. 12897240 10.1073/pnas.1631700100 PMC187889

[CIT0044] Mcnamara KB , Van Lieshout E, Simmons LW. 2014. The effect of maternal and paternal immune challenge on offspring immunity and reproduction in a cricket. J Evol Biol. 27(6):1020–1028. 24750259 10.1111/jeb.12376

[CIT0045] Mehlis M , Rick IP, Bakker TCM. 2015. Dynamic resource allocation between pre- and postcopulatory episodes of sexual selection determines competitive fertilization success. Proc R Soc B Biol Sc 282(1817):1–9. 10.1098/rspb.2015.1279 PMC4633866 26490787

[CIT0046] Milutinovic B , Kurtz J. 2016. Immune memory in invertebrates. Semin Immunol. 28(4):328–342. 27402055 10.1016/j.smim.2016.05.004

[CIT0047] Miyashita A , Lee TYM, Mcmillan LE, Easy R, Adamo SA. 2019. Immunity for nothing and the eggs for free: apparent lack of both physiological trade-offs and terminal reproductive investment in female crickets (*Gryllus texensis*). PLoS One. 14(5):e0209957. 31091239 10.1371/journal.pone.0209957 PMC6519836

[CIT0048] Moller AP , Petrie M. 2002. Condition dependence, multiple sexual signals, and immunocompetence in peacocks. Behav Ecol. 13(2):248–253.

[CIT0049] Nakagawa S. 2004. A farewell to Bonferroni: the problems of low statistical power and publication bias. Behav Ecol. 15(6):1044–1045.

[CIT0050] Nielsen ML , Holman L. 2012. Terminal investment in multiple sexual signals: immune-challenged males produce more attractive pheromones. Funct Ecol. 26(1):20–28.

[CIT0051] Parker G. 1998. Sperm competition and the evolution of ejaculates: towards a theory base. In: T. Birkhead, A. Møller, editors. Sperm competition and sexual selection. London: Academic Press. p. 3–54.

[CIT0052] Parker GA , Lessells CM, Simmons LW. 2013. Sperm competition games: a general model for precopulatory male-male competition. Evolution 67(1):95–109. 23289564 10.1111/j.1558-5646.2012.01741.x

[CIT0053] R Core Team. 2020. R: a language and environment for statistical computing. Vienna, Austria: R Foundation for Statistical Computing.

[CIT0054] Reaney LT , Knell RJ. 2010. Immune activation but not male quality affects female current reproductive investment in a dung beetle. Behav Ecol. 21(6):1367–1372.

[CIT0055] Rebar D , Bailey NW, Zuk M. 2009. Courtship song’s role during female mate choice in the field cricket *Teleogryllus oceanicus*. Behav Ecol. 20(6):1307–1314.

[CIT0056] RStudio Team. 2020. RStudio: integrated development for R. Boston (MA): RStudio, PBC.

[CIT0057] Rutkowski N-AJ , Foo YZ, Jones TM, Mcnamara KB. 2023. Age, but not an immune challenge, triggers terminal investment in the Pacific field cricket. Behavioural Ecology. 10.5061/dryad.3tx95x6m2. PMC10183208 37192922

[CIT0058] Sadd B , Holman L, Armitage H, Lock F, Marland R, Siva-Jothy MT. 2006. Modulation of sexual signalling by immune challenged male mealworm beetles (*Tenebrio molitor, L.*): evidence for terminal investment and dishonesty. J Evol Biol. 19(2):321–325. 16599907 10.1111/j.1420-9101.2005.01062.x

[CIT0059] Schaefer HM , Wilkinson DM. 2004. Red leaves, insects and coevolution: a red herring? Trends Ecol Evol 19(12):616–618. 16701323 10.1016/j.tree.2004.09.009

[CIT0060] Schwanz LE. 2008. Persistent effects of maternal parasitic infection on offspring fitness: implications for adaptive reproductive strategies when parasitized. Funct Ecol. 22(4):691–698.

[CIT0061] Shoemaker KL , Parsons NM, Adamo SA. 2006. Egg-laying behaviour following infection in the cricket *Gryllus texensis*. Can J Zool. 84(3):412–418.

[CIT0062] Simmons LW. 2001. Sperm competition and its evolutionary consequences in the insects. New Jersey, United States: Princeton University Press.

[CIT0063] Simmons LW. 2012. Resource allocation trade-off between sperm quality and immunity in the field cricket, *Teleogryllus oceanicus*. Behav Ecol. 23(1):168–173.

[CIT0064] Simmons LW , Beveridge M. 2011. Seminal fluid affects sperm viability in a cricket. PLoS One. 6(3):e17975. 21455309 10.1371/journal.pone.0017975 PMC3063794

[CIT0065] Simmons LW , Lupold S, Fitzpatrick JL. 2017. Evolutionary trade-off between secondary sexual traits and ejaculates. Trends Ecol Evol 32(12):964–976. 29050795 10.1016/j.tree.2017.09.011

[CIT0066] Simmons LW , Roberts B. 2005. Bacterial immunity traded for sperm viability in male crickets. Science. 309(5743):2031–2031. 16179472 10.1126/science.1114500

[CIT0067] Simmons LW , Tan YF, Millar AH. 2013a. Sperm and seminal fluid proteomes of the field cricket *Teleogryllus oceanicus*: identification of novel proteins transferred to females at mating. Insect Mol Biol. 22(1):115–130. 23211034 10.1111/imb.12007

[CIT0068] Simmons LW , Thomas ML, Simmons FW, Zuk M. 2013b. Female preferences for acoustic and olfactory signals during courtship: male crickets send multiple messages. Behav Ecol. 24(5):1099–1107.

[CIT0069] Simmons LW , Tinghitella RM, Zuk M. 2010. Quantitative genetic variation in courtship song and its covariation with immune function and sperm quality in the field cricket *Teleogryllus oceanicus*. Behav Ecol. 21(6):1330–1336.

[CIT0070] Sinervo B , Svensson E. 1998. Mechanistic and selective causes of life history trade-offs and plasticity. Oikos 83(3):432–442.

[CIT0071] Sohal RS. 1976. Aging changes in insect flight muscle. Gerontology. 22(4):317–333. 1269937 10.1159/000212146

[CIT0072] Stearns SC. 1989. Trade-offs in life-history evolution. Funct Ecol. 3(3):259–268.

[CIT0073] Sturm R. 2014. Comparison of sperm number, spermatophore size, and body size in four cricket species. J Orthoptera Res 23(1), 39–47, 9.

[CIT0074] Svensson E , Raberg L, Koch C, Hasselquist D. 1998. Energetic stress, immunosuppression and the costs of an antibody response. Funct Ecol. 12(6):912–919.

[CIT0075] Tanaka H , Sagisaka A, Fujita K, Kaneko Y, Imanishi S, Yamakawa M. 2009. Lipopolysaccharide elicits expression of immune-related genes in the silkworm, *Bombyx mori*. Insect Mol Biol. 18(1):71–75. 19196348 10.1111/j.1365-2583.2009.00851.x

[CIT0076] Tregenza T , Simmons LW, Wedell N, Zuk M. 2006. Female preference for male courtship song and its role as a signal of immune function and condition. Anim Behav. 72:809–818.

[CIT0077] Tregenza T , Wedell N. 1998. Benefits of multiple mates in the cricket *Gryllus bimaculatus*. Evolution 52(6):1726–1730. 28565303 10.1111/j.1558-5646.1998.tb02252.x

[CIT0078] Velando A , Drummond H, Torres R. 2006. Senescent birds redouble reproductive effort when ill: confirmation of the terminal investment hypothesis. Proc R Soc B Biol Sci 273(1593):1443–1448. 10.1098/rspb.2006.3480 PMC1560321 16777735

[CIT0079] Wedell N. 1997. Ejaculate size in bushcrickets: the importance of being large. J Evol Biol. 10(3):315–325.

[CIT0080] Wigby S , Sirot LK, Linklater JR, Buehner N, Calboli FCF, Bretman A, Wolfner MF, Chapman T. 2009. Seminal fluid protein allocation and male reproductive success. Curr Biol. 19(9):751–757. 19361995 10.1016/j.cub.2009.03.036 PMC2737339

[CIT0081] Williams GC. 1966. Natural selection, the costs of reproduction, and a refinement of lack’s principle. Am Nat. 100(916):687–690.

[CIT0082] Win AT , Kojima W, Ishikawa Y. 2013. Age-related male reproductive investment in courtship display and nuptial gifts in a moth, *Ostrinia scapulalis*. Ethology 119(4):325–334.

[CIT0083] Zera AJ , Harshman LG. 2001. The physiology of life history trade-offs in animals. Annu Rev Ecol Syst. 32:95–126.

[CIT0084] Zuk M , Rebar D, Scott SP. 2008. Courtship song is more variable than calling song in the field cricket *Teleogryllus oceanicus*. Anim Behav. 76:1065–1071.

