# Supplementary material for: Age, but not an immune challenge, triggers terminal investment in the Pacific field cricket, Teleogryllus oceanicus
Source: Behav Ecol. 2023 Mar 30;34(3):468–79. doi: 10.1093/beheco/arad021 (PMC10183208; doi:10.1093/beheco/arad021)
Supplement: arad021_suppl_Supplementary_Material [file arad021_suppl_supplementary_material.docx]

Supplementary material: Age, but not an immune challenge, triggers terminal investment in the Pacific field cricket, *Teleogryllus oceanicus*

Table S1. Summary of fit and loadings of PCA and mean ± standard errors (SE) for the nine song parameters of ‘young’ and ‘old’ males exposed to different doses of an immune challenge.

|  | Old | | | | Young | | | |  |  |  |
| --- | --- | --- | --- | --- | --- | --- | --- | --- | --- | --- | --- |
|  | Control | 0.1% | 0.5% | 1% | Control | 0.1% | 0.5% | 1% | PC1 | PC2 | PC3 |
| Eigenvalues | |  |  |  |  |  |  |  | 2.56 | 2.08 | 1.77 |
| % variance explained | | |  |  |  |  |  |  | 28.5 | 23.1 | 19.6 |
| *n* | 12 | 11 | 11 | 11 | 14 | 10 | 7 | 10 |  |  |  |
| CD | 0.49 ± 0.03 | 0.47 ± 0.02 | 0.47 ± 0.04 | 0.48 ± 0.02 | 0.47 ± 0.03 | 0.51 ± 0.03 | 0.45 ± 0.03 | 0.4 ± 0.02 | 0.77 | 0.45 | -0.35 |
| CPI | 0.04 ± 0.00 | 0.04 ± 0.00 | 0.04 ± 0.01 | 0.04 ± 0.00 | 0.04 ± 0.00 | 0.04 ± 0.00 | 0.04 ± 0.00 | 0.04 ± 0.00 | -0.23 | 0.10 | 0.64 |
| CPD | 0.03 ± 0.00 | 0.03 ± 0.00 | 0.03 ± 0.00 | 0.03 ± 0.00 | 0.03 ± 0.00 | 0.03 ± 0.00 | 0.03 ± 0.00 | 0.03 ± 0.00 | 0.27 | -0.62 | -0.34 |
| CPN | 7.5 ± 0.39 | 7.11 ± 0.36 | 6.93 ± 0.54 | 7.4 ± 0.27 | 7.61 ± 0.53 | 8.15 ± 0.46 | 7.11 ± 0.48 | 7.41 ± 0.51 | 0.68 | 0.57 | -0.37 |
| CTI | 0.1 ± 0.02 | 0.21 ± 0.11 | 0.08 ± 0.01 | 0.12 ± 0.02 | 0.14 ± 0.03 | 0.17 ± 0.07 | 0.19 ± 0.09 | 0.11 ± 0.01 | -0.42 | 0.50 | -0.01 |
| TD | 2.61 ± 0.17 | 3.30 ± 0.31 | 2.71 ± 0.36 | 2.52 ± 0.21 | 2.88 ± 0.27 | 3.15 ± 0.31 | 2.97 ± 0.30 | 1.39 ± 0.46 | 0.46 | -0.64 | -0.34 |
| TPI | 0.02 ± 0.00 | 0.01 ± 0.00 | 0.01 ± 0.00 | 0.01 ± 0.00 | 0.01 ± 0.00 | 0.02 ± 0.00 | 0.01 ± 0.00 | 0.01 ± 0.00 | 0.02 | 0.70 | -0.10 |
| TPD | 0.02 ± 0.00 | 0.02 ± 0.00 | 0.02 ± 0.00 | 0.02 ± 0.00 | 0.02 ± 0.00 | 0.02 ± 0.00 | 0.02 ± 0.00 | 0.02 ± 0.00 | 0.46 | -0.64 | -0.34 |
| TPN | 77.16 ± 6.87 | 97.41 ± 11.33 | 79.03 ± 9.80 | 78.00 ± 7.07 | 88.71 ± 9.10 | 85.45 ± 9.16 | 83.11 ± 9.96 | 77.27 ± 11.84 | 0.67 | -0.08 | 0.69 |

## CD = chirp duration, CPI = chirp pulse interval, CPD = chirp pulse duration, CPN = Chirp pulse number, CTI = chirp-trill interval, TD = trill duration, TPI = trill pulse interval, TPD = trill pulse duration, TPN = Trill pulse number.

Table S2. Sample sizes for all models presented in the main paper.

|  | Control | 0.1% LPS | 0.5% LPS | 1% LPS |
| --- | --- | --- | --- | --- |
| *Likelihood of calling / Likelihood of mating* |  |  |  |  |
| Young | 23 | 17 | 17 | 16 |
| Old | 22 | 19 | 20 | 19 |
| *Latency to calling* |  |  |  |  |
| Young | 16 | 10 | 11 | 11 |
| Old | 15 | 12 | 15 | 11 |
| *Calls within bout one* |  |  |  |  |
| Young | 11 | 10 | 7 | 8 |
| Old | 11 | 10 | 12 | 9 |
| No. of calling bouts / Bout length / No. of calls in each bout |  |  |  |  |
| Young | 23 | 22 | 17 | 18 |
| Old | 20 | 29 | 31 | 24 |
| Inter-bout interval |  |  |  |  |
| Young | 12 | 12 | 10 | 10 |
| Old | 9 | 20 | 19 | 15 |
| Latency to mating |  |  |  |  |
| Young | 14 | 8 | 8 | 9 |
| Old | 15 | 11 | 10 | 6 |
| No. of ejaculates transferred |  |  |  |  |
| Young | 24 | 16 | 17 | 16 |
| Old | 23 | 19 | 19 | 19 |
| Likelihood of laying |  |  |  |  |
| Young | 14 | 8 | 7 | 8 |
| Old | 16 | 11 | 9 | 6 |
| No. of eggs laid / Fertility |  |  |  |  |
| Young | 9 | 3 | 4 | 6 |
| Old | 11 | 6 | 5 | 6 |
| Weight of spermatophore |  |  |  |  |
| Young | 14 | 11 | 15 | 10 |
| Old | 18 | 14 | 15 | 14 |
| Longevity |  |  |  |  |
| Young | 23 | 16 | 20 | 25 |
| Old | 23 | 20 | 22 | 27 |

Table S3-S9. Model summaries for all analysis presented in the main paper but excluding the 1% LPS dose. We present this data to demonstrate that, despite the apparent selection imposed by this highest dose, removing it from analysis did not have a demonstrable impact on our key findings.

Table S3. Summary of fit and loadings of PCA and mean ± standard errors (SE) for the nine song parameters of ‘young’ and ‘old’ males exposed to different doses of an immune challenge (excluding the 1% LPS dose).

|  | Old | | | Young | | |  | | |
| --- | --- | --- | --- | --- | --- | --- | --- | --- | --- |
|  | Control | 0.1% | 0.5% | Control | 0.1% | 0.5% | PC1 | PC2 | PC3 |
| Eigenvalues | |  |  |  |  |  | 2.55 | 2.10 | 1.87 |
| % of variance explained | |  |  |  |  |  | 28.3 | 23.3 | 20.7 |
| *n* | 12 | 11 | 11 | 14 | 10 | 7 |  |  |  |
| CD | 0.49 ± 0.03 | 0.47 ± 0.02 | 0.47 ± 0.04 | 0.47 ± 0.03 | 0.51 ± 0.03 | 0.45 ± 0.03 | 0.70 | 0.64 | -0.16 |
| CPI | 0.04 ± 0.00 | 0.04 ± 0.002 | 0.04 ± 0.01 | 0.04 ± 0.00 | 0.04 ± 0.00 | 0.04 ± 0.00 | -0.29 | -0.05 | 0.66 |
| CPD | 0.03 ± 0.00 | 0.03 ± 0.00 | 0.03 ± 0.001 | 0.03 ± 0.00 | 0.03 ± 0.00 | 0.03 ± 0.00 | 0.38 | -0.42 | 0.53 |
| CPN | 7.50 ± 0.39 | 7.11 ± 0.36 | 6.93 ± 0.54 | 7.61 ± 0.53 | 8.15 ± 0.46 | 7.11 ± 0.48 | 0.59 | 0.74 | -0.14 |
| CTI | 0.10 ± 0.02 | 0.21 ± 0.11 | 0.08 ± 0.01 | 0.14 ± 0.03 | 0.17 ± 0.07 | 0.19 ± 0.09 | -0.53 | 0.43 | 0.04 |
| TD | 2.61 ± 0.17 | 3.30 ± 0.31 | 2.71 ± 0.36 | 2.88 ± 0.27 | 3.15 ± 0.31 | 2.97 ± 0.30 | 0.68 | -0.14 | 0.65 |
| TPI | 0.02 ± 0.00 | 0.01 ± 0.00 | 0.01 ± 0.00 | 0.01 ± 0.001 | 0.02 ± 0.00 | 0.01 ± 0.00 | 0.001 | 0.71 | 0.12 |
| TPD | 0.02 ± 0.00 | 0.02 ± 0.00 | 0.02 ± 0.00 | 0.02 ± 0.00 | 0.02 ± 0.00 | 0.02 ± 0.00 | 0.60 | 0.44 | -0.44 |
| TPN | 77.16 ± 6.87 | 97.41 ± 11.33 | 79.03 ± 9.80 | 88.71 ± 9.10 | 85.45 ± 9.16 | 83.11 ± 9.96 | 0.61 | -0.25 | 0.69 |

Table S4. Model summaries for the effect of LPS dose (excluding the 1% LPS dose) and male age weight on the three principal components. The term after the ± is standard error. Parameter estimates for each level of non-significant LPS dose x age interactions are not reported.

|  | PC1 | | |  | PC2 | | |  | PC3 | | |
| --- | --- | --- | --- | --- | --- | --- | --- | --- | --- | --- | --- |
|  | Parameter estimate | Statistic | P |  | Parameter estimate | Statistic | P |  | Parameter estimate | Statistic | P |
| Intercept | -0.29 ± 0.41 | t = -0.72 | 0.47 |  | 0.16 ± 0.37 | t = -0.43 | 0.67 |  | -0.07 ± 0.35 | t =-0.21 | 0.84 |
| Age |  | F_1,60_ = 0.52 | 0.47 |  |  | F_1,60_ = 0.05 | 0.82 |  |  | F_1,60_ = 0.37 | 0.55 |
| Age (young) | 0.35 ± 0.48 | t = 0.72 | 0.47 |  | -0.10 ± 0.43 | t = -0.22 | 0.82 |  | 0.25 ± 0.41 | t =0.61 | 0.55 |
| LPS |  | F_2,60_ = 0.43 | 0.65 |  |  | F_2,60_ = 1.00 | 0.37 |  |  | F_2,58_ = 0.58 | 0.56 |
| LPS (0.1) | 0.35 ± 0.48 | t = 0.72 | 0.47 |  | 0.08 ± 0.43 | t = 0.18 | 0.86 |  | 0.13 ± 0.41 | t =0.33 | 0.74 |
| LPS (0.5) | -0.008 ± 0.50 | t = -0.02 | 0.99 |  | 0.61 ± 0.45 | t = -1.35 | 0.18 |  | -0.33 ± 0.43 | t =-0.78 | 0.44 |
| Relative male weight | -0.32 ± 0.24 | F_2,60_ = 1.80 | 0.19 |  | -0.17 ± 0.22 | F_1,60_ = 0.60 | 0.44 |  | -0.01 ± 0.21 | F_1,60_ = 0.005 | 0.95 |
| LPS x Age |  | F_2,58_ = 0.27 | 0.76 |  |  | F_2,58_ = 0.89 | 0.42 |  |  | F_2,58_ = 0.11 | 0.90 |
| LPS (0.1) x Age (young) |  | t = 0.22 | 0.83 |  |  | t = -1.31 | 0.26 |  |  | t =-0.16 | 0.87 |
| LPS (0.5) x Age (young) |  | t = -0.56 | 0.58 |  |  | t = -0.72 | 0.48 |  |  | t =0.33 | 0.74 |

Table S5. The effect of age and LPS dose (excluding the 1% dose) on male calling behaviour. Bolded values are significant, with P < 0.05. The number in parentheses represents the exponent of the transformation. The term after the ± is standard error. Parameter estimates for each level of non-significant LPS dose x age interactions are not reported.

|  | Likelihood of calling | | |  | Latency to calling (^0.04) | | |
| --- | --- | --- | --- | --- | --- | --- | --- |
|  | Parameter estimate | Statistic | P |  | Parameter estimate | Statistic | P |
| Intercept | 2.17 ± 0.55 | z = 3.94 | **0.00008** |  | 1.08 ± 0.006 | t = 166.91 | **0.00** |
| Age |  | χ^2^_1_ = 0.05 | 0.83 |  |  | F_1,74_ = 0.95 | 0.33 |
| Age (young) | -0.11 ± 0.50 | z = -0.22 | 0.83 |  | 0.007 ± 0.007 | t = 0.97 | 0.33 |
| LPS |  | χ^2^_2_ = 1.62 | 0.44 |  |  | F_2,74_ = 3.34 | **0.04** |
| LPS (0.1) | -0.77 ± 0.63 | z = -1.21 | 0.22 |  | -0.02 ± 0.008 | t = -2.05 | **0.04** |
| LPS (0.5) | -0.55 ± 0.64 | z = -0.86 | 0.39 |  | 0.004 ± 0.008 | t = 0.52 | 0.61 |
| Relative male weight | -0.37 ± 0.27 | χ^2^_1_ = 1.96 | 0.16 |  | -0.001 ± 0.003 | F_1,74_ = 0.08 | 0.77 |
| LPS x Age |  | χ^2^_2_ = 0.91 | 0.63 |  |  | F_1,72_ = 0.11 | 0.89 |
| LPS (0.1) x Age (young) |  | z = 0.70 | 0.48 |  |  | t = -0.21 | 0.84 |
| LPS (0.5) x Age (young) |  | z = -0.12 | 0.90 |  |  | t = -0.48 | 0.63 |

Table S6. The effect of age and LPS dose (excluding the 1% dose) on male calling bouts. Bolded values are significant, with P < 0.05. The number in parentheses represents the exponent of the transformation. The term after the ± is standard error.

|  | Calls within bout one | | |  | No. of calling bouts (^0.56) | | |  | Bout length (^0.04) | | |
| --- | --- | --- | --- | --- | --- | --- | --- | --- | --- | --- | --- |
|  | Parameter estimate | Statistic | P |  | Parameter estimate | Statistic | P |  | Parameter estimate | Statistic | P |
| Intercept | 1.90 ± 0.12 | t = 16.23 | **0.00** |  | 1.47 ± 0.10 | t = 15.46 | **0.00** |  | 1.12 ± 0.01 | t = 109.64 | **0.00** |
| Age |  | χ^2^_1_ = 11.35 | **0.003** |  |  | F_1,137_ = 1.61 | 0.21 |  |  | F_1,137_ = 0.05 | 0.83 |
| Age (young) | 0.40 ± 0.15 | t = 2.65 | **0.008** |  | 0.32 ± 0.13 | t = 2.47 | **0.01** |  | 0.002 ± 0.008 | t = 0.22 | 0.83 |
| LPS |  | χ^2^_2_ = 7.16 | **0.007** |  |  | F_2,137_ = 0.12 | 0.90 |  |  | F_2,137_ = 0.22 | 0.80 |
| LPS (0.1) | 0.16 ± 0.16 | t = 1.01 | 0.31 |  | 0.43 ± 0.12 | t = 3.54 | **0.0006** |  | -0.005 ± 0.009 | t = -0.50 | 0.62 |
| LPS (0.5) | 0.005 ± 0.16 | t = 0.43 | 0.67 |  | 0.33 ± 0.12 | t = 2.72 | **0.007** |  | -0.006 ± 0.01 | t = -0.64 | 0.52 |
| Relative male weight | 0.01 ± 0.05 | χ^2^_1_ = 0.07 | 0.79 |  | 0.04 ± 0.04 | F_1,137_ = 0.95 | 0.33 |  | 0.002 ± 0.004 | F_2,137_ = 0.34 | 0.56 |
| Bout number | NA | NA | NA |  | NA | NA | NA |  | -0.003 ± 0.004 | F_2,137_ = 0.53 | 0.47 |
| LPS x Age |  | χ^2^_2_ = 9.18 | **0.01** |  |  | F_2,135_ = 3.73 | **0.03** |  |  | F_2,135_ = 0.59 | 0.56 |
| LPS (0.1) x Age (young) | -0.64 ± 0.23 | t = 2.82 | **0.005** |  | -0.47 ± 0.17 | t = -2.71 | **0.008** |  |  | t = -0.56 | 0.58 |
| LPS (0.5) x Age (young) | -0.51 ± 0.24 | t = -2.13 | **0.03** |  | -0.31 ± 0.18 | t = -1.72 | 0.09 |  |  | t = 0.50 | 0.62 |

Table S7. The effect of age and LPS dose (excluding the 1% dose) on male calling bouts, continued. Bolded values are significant, with P < 0.05. The number in parentheses represents the exponent of the transformation. The term after the ± is standard error. Parameter estimates for each level of non-significant LPS dose x age interactions are not reported.

|  | Calls within each bout (^0.04) | | |  | Inter-bout interval (^0.04) | | |
| --- | --- | --- | --- | --- | --- | --- | --- |
|  | Parameter estimate | Statistic | P |  | Parameter estimate | Statistic | P |
| Intercept | 1.06 ± 0.009 | t = 118.23 | **0.00** |  | 1.13 ± 0.009 | t = 119.12 | **0.00** |
| Age |  | F_1,137_ = 0.06 | 0.81 |  |  | F_1,76_ = 6.43 | **0.01** |
| Age (young) | 0.001 ± 0.007 | t =0.24 | 0.81 |  | -0.02 ± 0.007 | t = -2.54 | **0.01** |
| LPS |  | F_2,137_ = 0.41 | 0.66 |  |  | F_2,76_ = 1.59 | 0.21 |
| LPS (0.1) | -0.007 ± 0.008 | t = -0.90 | 0.37 |  | -0.01 ± 0.008 | t = -1.75 | 0.08 |
| LPS (0.5) | -0.003 ± 0.008 | t = -0.39 | 0.70 |  | -0.007 ± 0.008 | t = -0.81 | 0.42 |
| Relative male weight | 0.004 ± 0.004 | F_1,137_ = 1.22 | 0.27 |  | -0.008 ± 0.003 | F_1,76_ = 4.70 | **0.03** |
| Bout number | -0.004 ±0.003 | F_1,137_ = 1.08 | 0.19 |  | -0.006 ± 0.004 | F_1,76_=3.02 | 0.09 |
| Age x LPS |  | F_1,135_ = 0.02 | 0.98 |  |  | F_1,74_ = 0.07 | 0.93 |
| LPS (0.1) x Age (young) |  | t = -0.11 | 0.91 |  |  | t = -0.25 | 0.80 |
| LPS (0.5) x Age (young) |  | t = 0.07 | 0.94 |  |  | t = 0.09 | 0.93 |

Table S8. The effect of male age and LPS dose (excluding the 1% dose) on male mating behaviour. Bolded values are significant, with P < 0.05. The number in parentheses represents the exponent of the transformation. The term after the ± is standard error. Second female refers to if a second female was required (1), or not (0). Parameter estimates for each level of non-significant LPS dose x age interactions are not reported.

|  | Likelihood of mating | | |  | Latency to mating (^0.48) | | |  | Number of ejaculates transferred (^1.16) | | |
| --- | --- | --- | --- | --- | --- | --- | --- | --- | --- | --- | --- |
|  | Parameter estimate | Statistic | P |  | Parameter estimate | Statistic | P |  | Parameter estimate | Statistic | P |
| Intercept | -0.17 ± 0.36 | z = -0.48 | 0.63 |  | 3.53 ± 1.25 | t = 2.83 | **0.006** |  | 1.57 ± 0.13 | t = 11.76 | **0.000** |
| Age |  | χ^2^_1_ = 1.05 | 0.59 |  |  | F_1,58_ = 0.85 | 0.36 |  |  | F_1,113_ = 1.44 | 0.23 |
| Age (young) | -0.13 ± 0.38 | z = -0.34 | 0.52 |  | 0.19 ± 0.21 | t = 0.92 | 0.40 |  | -0.17 ± 0.14 | t = -1.20 | 0.23 |
| LPS |  | χ^2^_2_ = 0.11 | 0.73 |  |  | F_2,58_ = 0.33 | 0.72 |  |  | F_2,113_ = 0.01 | 0.99 |
| LPS (0.1) | -0.30 ± 0.46 | z = -0.65 | 0.52 |  | -0.19 ± 0.25 | t = -0.76 | 0.45 |  | -0.03 ± 0.17 | t = -0.15 | 0.88 |
| LPS (0.5) | -0.46 ± 0.47 | z = -1.00 | 0.32 |  | -1.48 ± 1.33 | t = -1.11 | 0.27 |  | -0.007 ± 0.17 | t = -0.04 | 0.97 |
| Relative male weight | -0.22 ± 0.20 | χ^2^_1_ = 1.17 | 0.28 |  | -0.04 ± 0.10 | F_1,58_ = 0.13 | 0.72 |  | -0.12 ± 0.07 | F_1,113_ = 2.70 | 0.10 |
| Female weight | NA | NA | NA |  | -0.55 ± 1.02 | F_1,58_ = 0.29 | 0.59 |  | NA | NA | NA |
| Female age | NA | NA | NA |  | -0.05 ± 0.09 | F_1,58_ = 0.24 | 0.62 |  | NA | NA | NA |
| Second female (0/1) | NA | NA | NA |  | -0.69 ± 0.23 | F_1,58_ = 12.90 | **0.0007** |  | NA | NA | NA |
| LPS x Age |  | χ^2^_2_ = 0.70 | 0.71 |  |  | F_2,56_ = 0.43 | 0.65 |  |  | F_2,111_ = 0.94 | 0.39 |
| LPS (0.1) x Age (young) |  | z = -0.53 | 0.60 |  |  | t = -0.41 | 0.68 |  |  | t = -0.26 | 0.79 |
| LPS (0.5) x Age (young) |  | 0.36 | 0.72 |  |  | t = -0.71 | 0.48 |  |  | t = 1.11 | 0.27 |

Table S9. The effect of male age and LPS dose (excluding the 1% dose) on female reproductive output. The number in parentheses represents the exponent of the transformation. The term after the ± is standard error. Parameter estimates for each level of non-significant LPS dose x age interactions are not reported.

|  | Likelihood of laying eggs | | |  | Number of eggs laid (^0.8) | | |  | Fertility (proportion of eggs hatched) | | |
| --- | --- | --- | --- | --- | --- | --- | --- | --- | --- | --- | --- |
|  | Parameter estimate | Statistic | P |  | Parameter estimate | Statistic | P |  | Parameter estimate | Statistic | P |
| Intercept | -3.91 ± 3.53 | z = -1.11 | 0.27 |  | 134.01 ± 46.40 | t = 2.23 | **0.03** |  | 1.41 ± 4.71 | z = 0.30 | 0.77 |
| Age |  | χ^2^_1_ = 0.04 | 0.83 |  |  | F_1,22_ = 0.00 | 0.93 |  |  | χ^2^_1_ = 4.57 | 0.10 |
| Age (young) | -0.12 ± 0.56 | z =-0.21 | 0.83 |  | 1.20 ± 7.58 | t = 0.16 | 0.87 |  | -0.83 ± 0.67 | z = -1.24 | 0.23 |
| LPS |  | χ^2^_2_ = 3.75 | 0.15 |  |  | F_2,22_ = 0.14 | 0.87 |  |  | χ^2^_2_ = 1.59 | 0.20 |
| LPS (0.1) | -1.32 ± 0.71 | z =-1.87 | **0.06** |  | 2.81 ± 9.65 | t = 0.29 | 0.77 |  | 1.69 ± 0.97 | z = 1.74 | 0.1 |
| LPS (0.5) | -0.59 ± 0.69 | z =-0.85 | 0.39 |  | 2.18 ± 8.97 | t = 0.24 | 0.81 |  | 1.16 ± 0.70 | z = 1.67 | 0.11 |
| Relative male weight | -0.67 ± 0.29 | χ^2^_1_ = 6.03 | **0.01** |  | 8.46 ± 4.07 | F_1,22_ = 5.19 | **0.03** |  | 0.36 ± 0.45 | χ^2^_1_ = 0.69 | 0.40 |
| Female weight | 5.39 ± 2.79 | χ^2^_1_ = 0.31 | 0.58 |  | -57.53 ± 35.67 | F_1,22_ = 3.30 | 0.09 |  | -0.13 ± 0.34 | χ^2^_1_ = 0.25 | 0.62 |
| Female age | 0.15 ± 0.26 | χ^2^_1_ = 4.15 | **0.04** |  | -6.06 ± 3.25 | F_1,22_ = 3.17 | 0.08 |  | -1.85 ± 3.71 | χ^2^_1_ = 0.15 | 0.70 |
| LPS x Age |  | χ^2^_2_ = 0.76 | 0.69 |  |  | F_2,20_ = 0.26 | 0.78 |  |  | χ^2^_2_ = 3.41 | 0.18 |
| LPS (0.1) x Age (young) |  | z =-0.20 | 0.84 |  |  | t = 0.70 | 0.49 |  |  | z = 0.91 | 0.38 |
| LPS (0.5) x Age (young) |  | z =0.69 | 0.49 |  |  | t = 0.38 | 0.71 |  |  | z = 0.08 | 0.94 |

Table S10. The influence of male age and LPS dose (excluding the 1% dose) on spermatophore weight (mg) and longevity. Bolded values are significant, with P < 0.05. The number in parentheses represents the exponent of the transformation. The term after the ± is standard error. Parameter estimates for each level of non-significant LPS dose x age interactions are not reported.

|  | Weight of spermatophore | | |  | Male longevity (^0.04) | | |
| --- | --- | --- | --- | --- | --- | --- | --- |
|  | Parameter  estimates (x10^-4^) | Statistic | P |  | Parameter estimates | Statistic | P |
| Intercept | 20.0 ± 0.7 | t = 26.28 | **0.00** |  | 1.15 ± 0.005 | t = 238.76 | **0.00** |
| Age |  | F_1,81_ = 8.06 | **0.006** |  |  | F_1,11 2_= 17.90 | **0.00005** |
| Age (young) | -1.0 ± 0.5 | t = -2.84 | **0.006** |  | -0.01 ± 0.003 | t = 4.23 | **0.00005** |
| LPS |  | F_2,81_ = 1.65 | 0.20 |  |  | F_2,112_ = 0.94 | 0.39 |
| LPS (0.1) | 0.5 ± 0.7 | t = 0.80 | 0.43 |  | -0.006 ± 0.004 | t = -1.37 | 0.17 |
| LPS (0.5) | -0.7 ± 0.6 | t = -1.05 | 0.30 |  | -0.003 ± 0.004 | t = -0.66 | 0.51 |
| Relative male weight | 0.9 ± 0.3 | F_1,81_ = 9.56 | **0.003** |  | 0.004 ± 0.002 | F_1,112_ = 4.37 | **0.04** |
| Number of prior matings | 1.0 ± 0.6 | F_1,81_ = 0.05 | 0.80 |  | 0.004 ± 0.003 | F_1,112_ = 2.43 | 0.12 |
| LPS x Age |  | F_2,79_ = 1.03 | 0.36 |  |  | F_2,110_ = 1.11 | 0.33 |
| LPS (0.1) x Age (young) |  | t = -0.45 | 0.65 |  |  | t = -1.34 | 0.18 |
| LPS (0.5) x Age (young) |  | t = -1.42 | 0.16 |  |  | t = 0.03 | 0.97 |
